# Supplementary figures and images for: In situ X-ray-assisted electron microscopy staining for large biological samples
Source: eLife. 2022 Oct 20;11:e72147. doi: 10.7554/eLife.72147 (PMC9665849; doi:10.7554/eLife.72147)

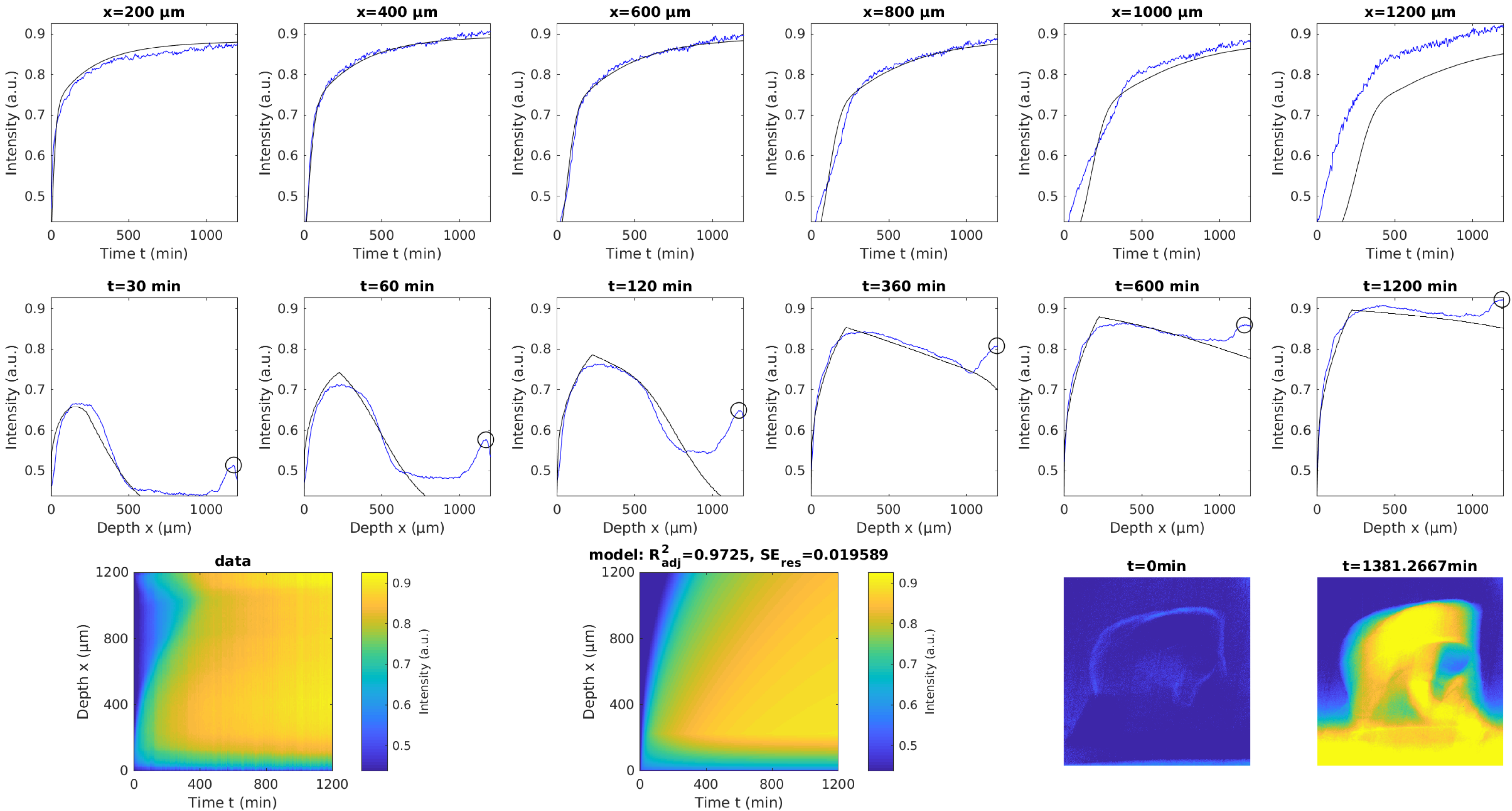

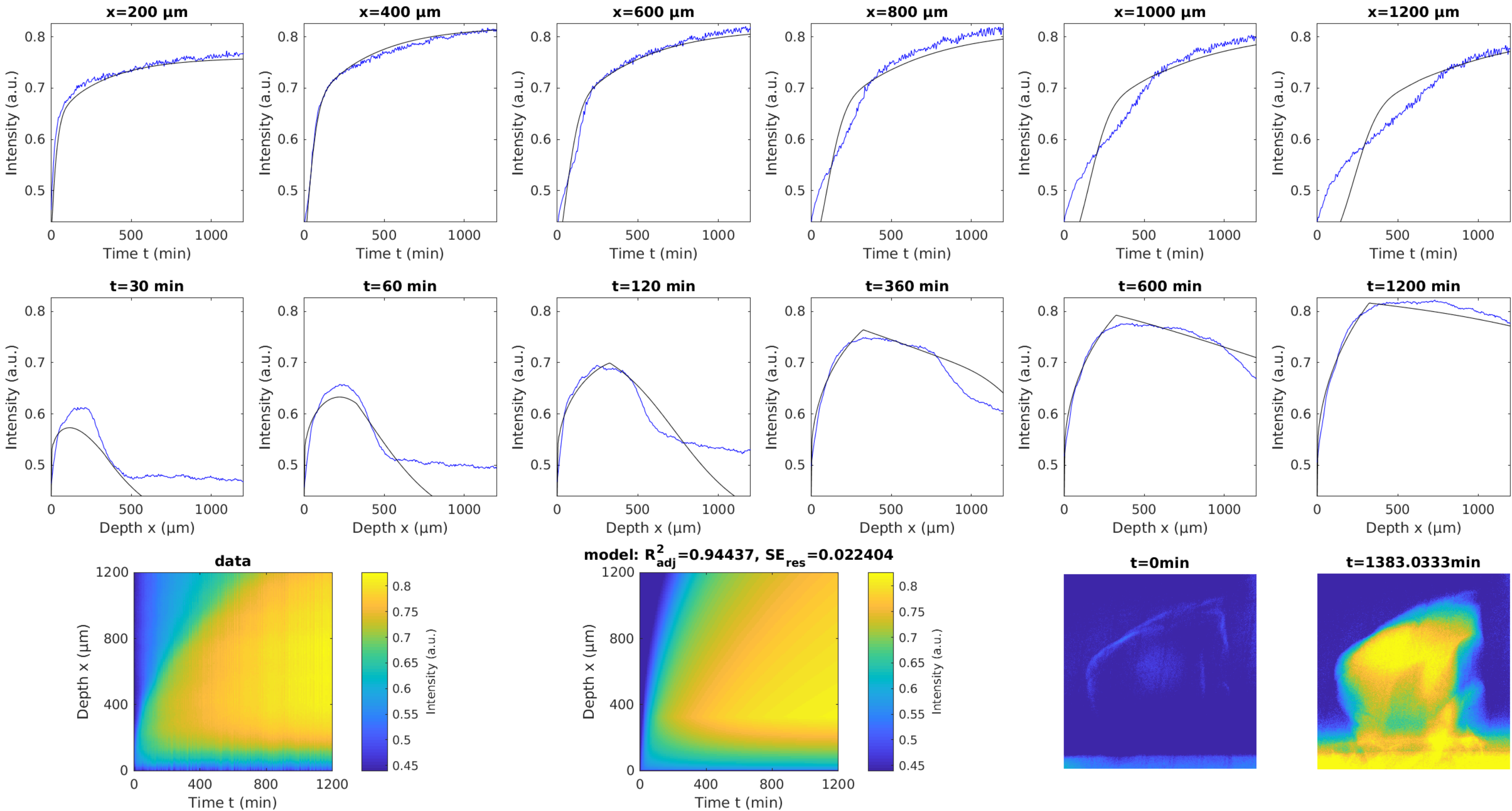

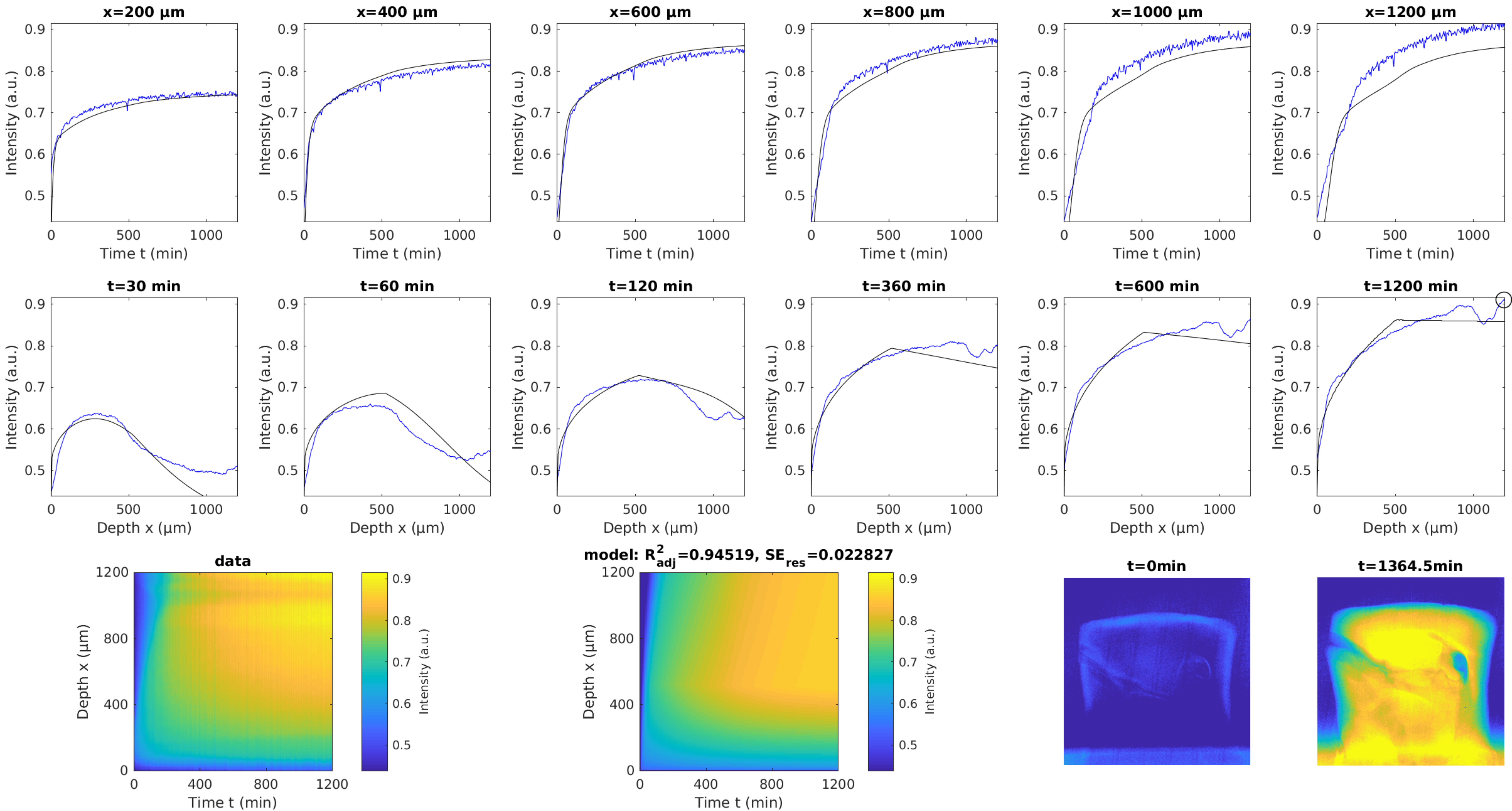

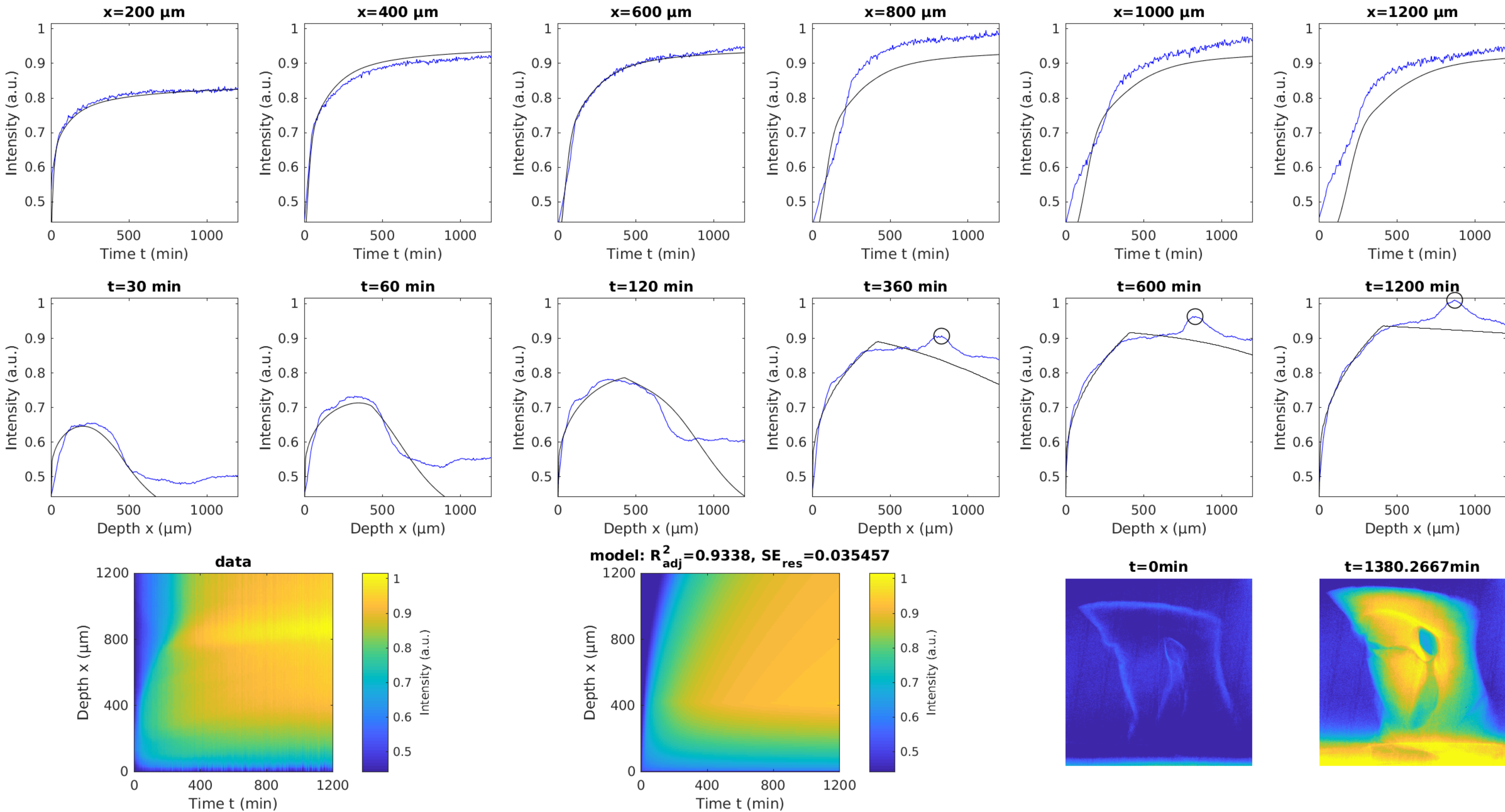

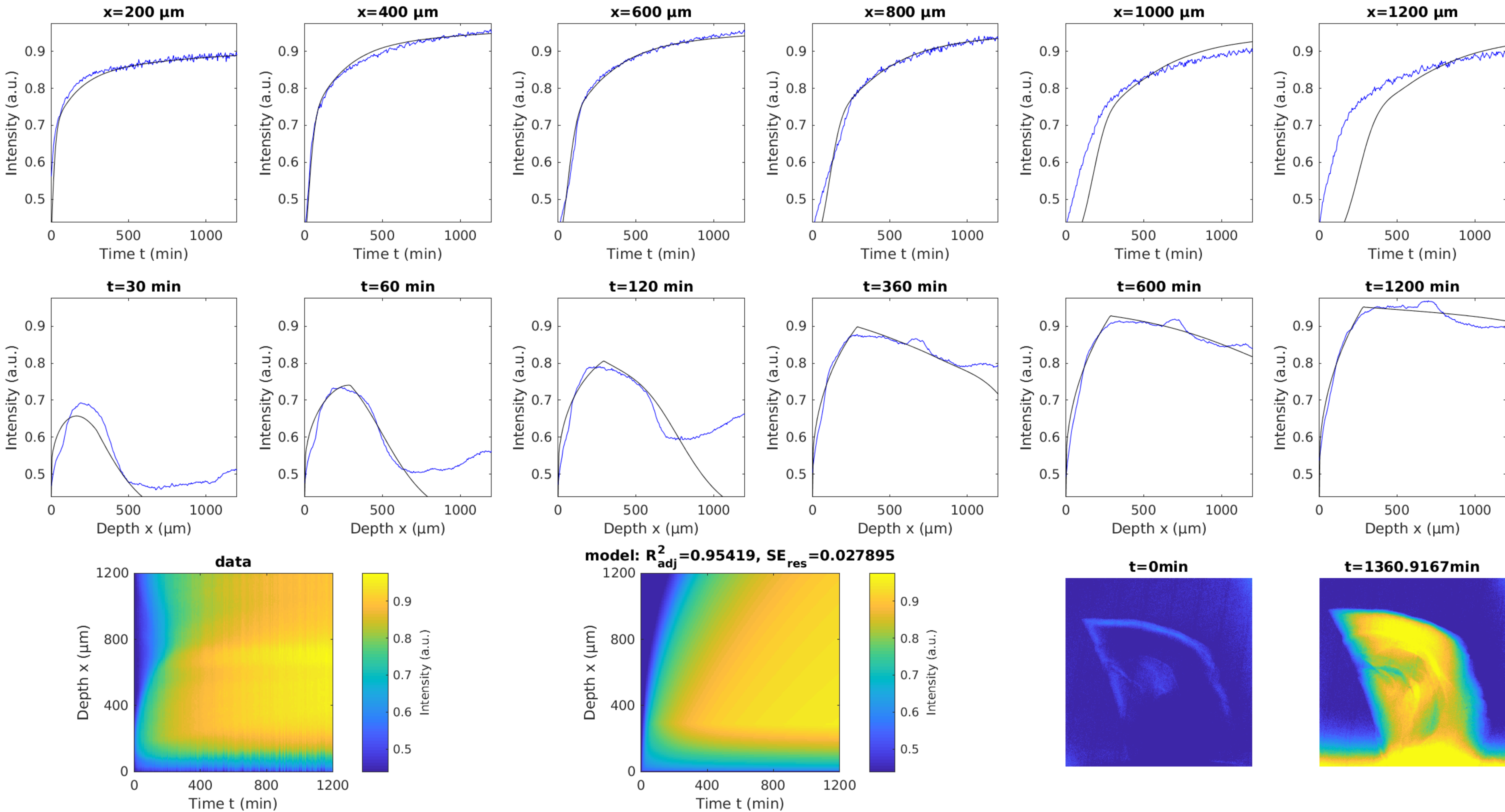

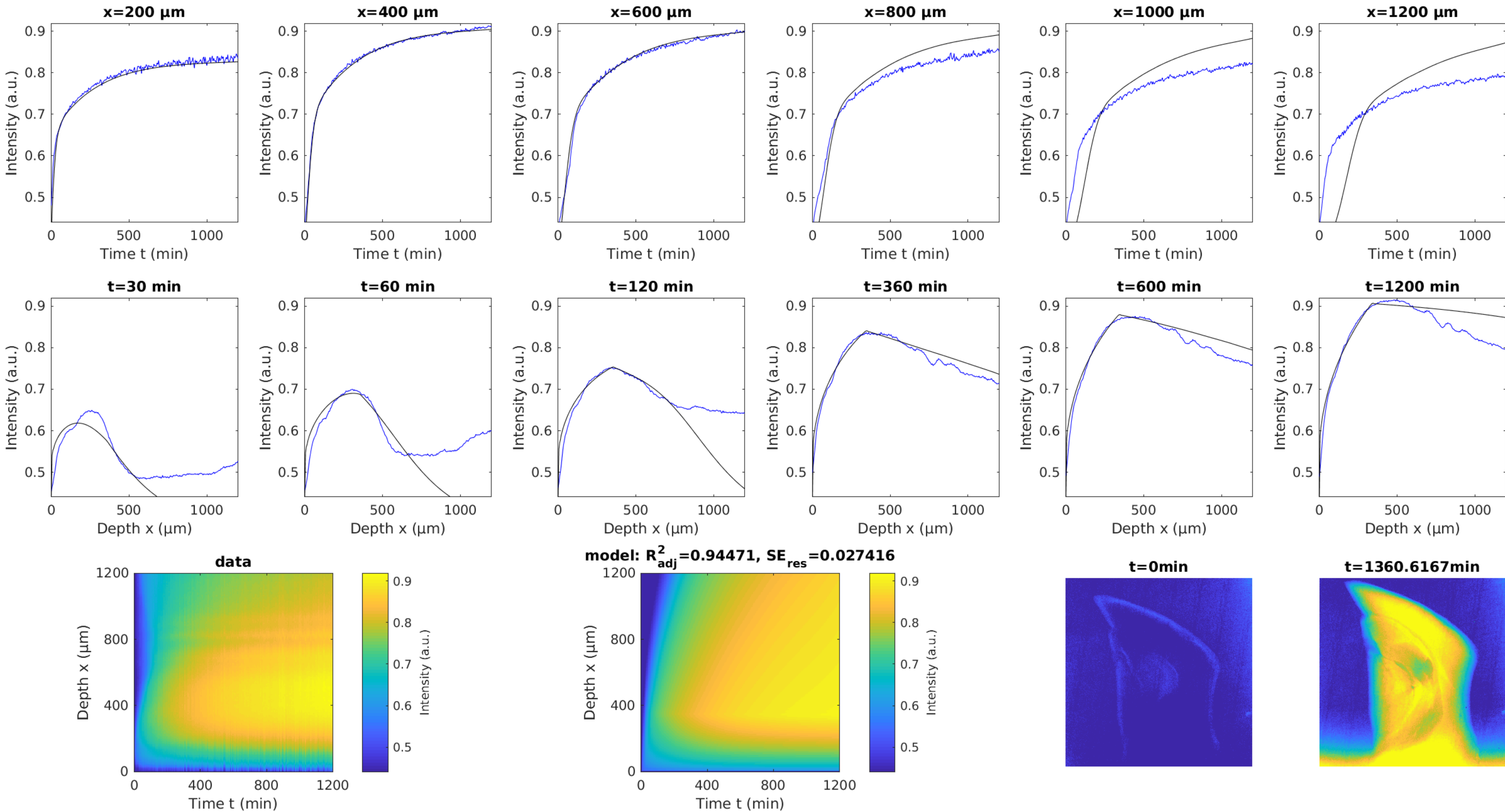

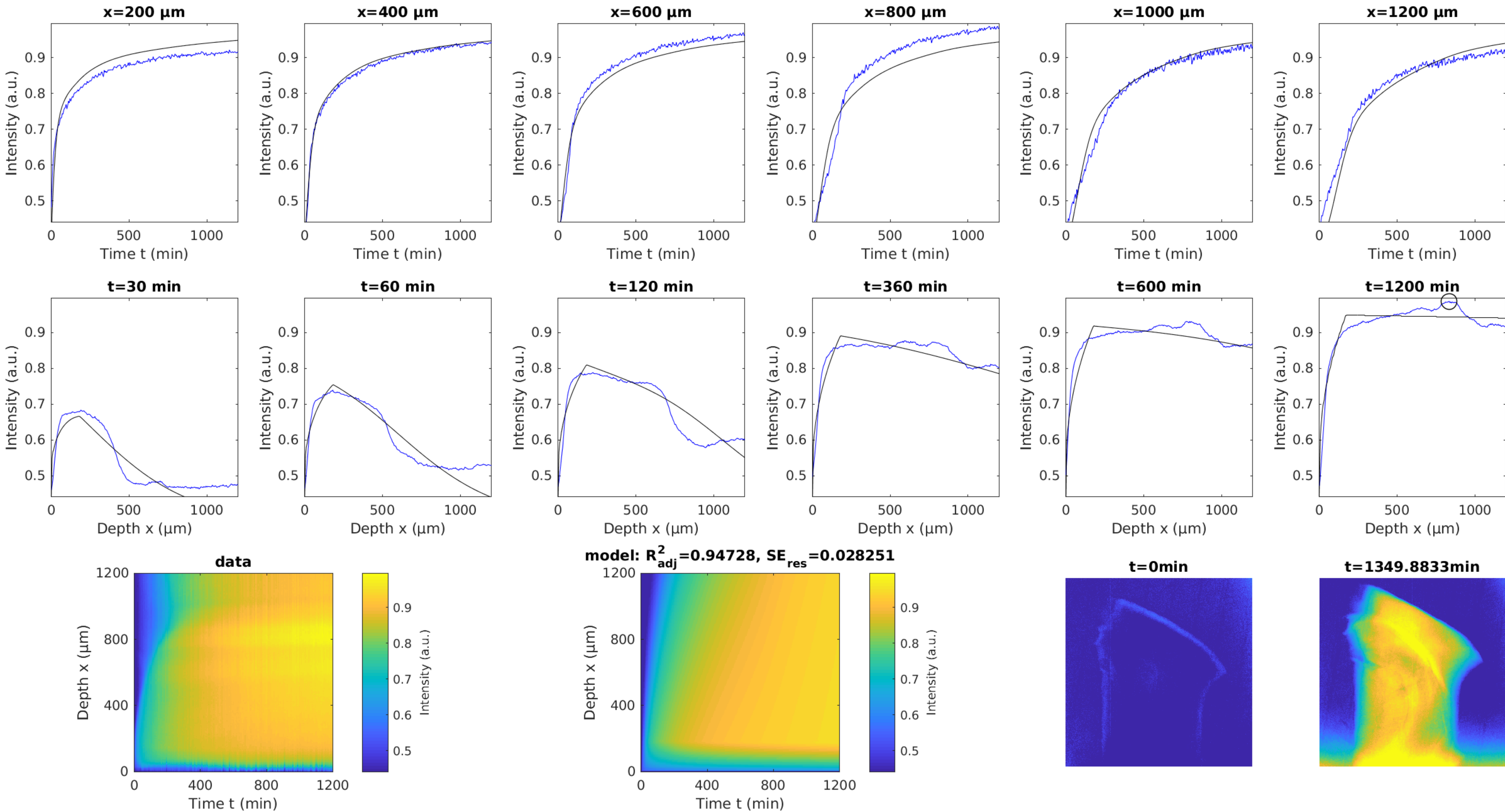

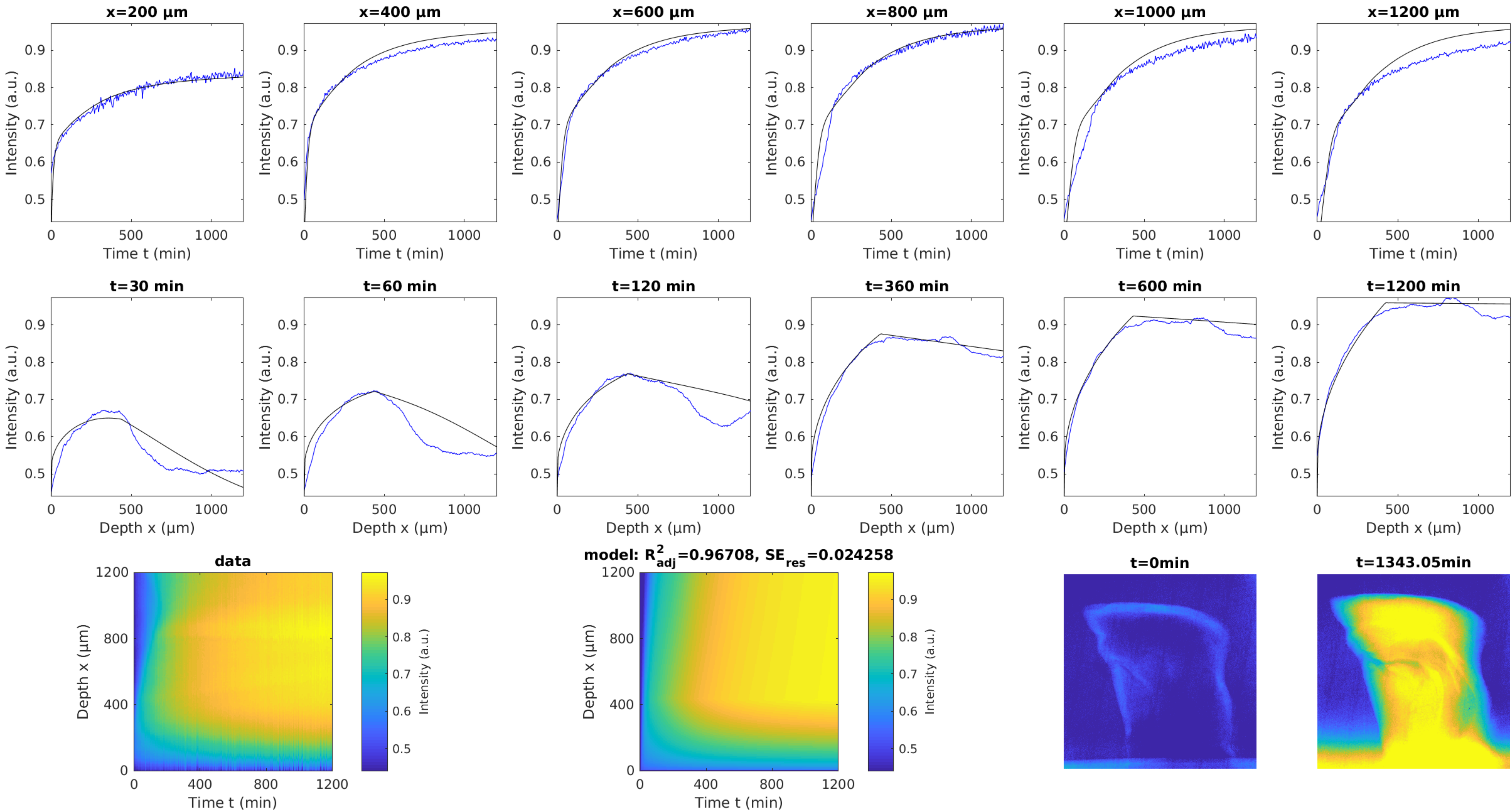

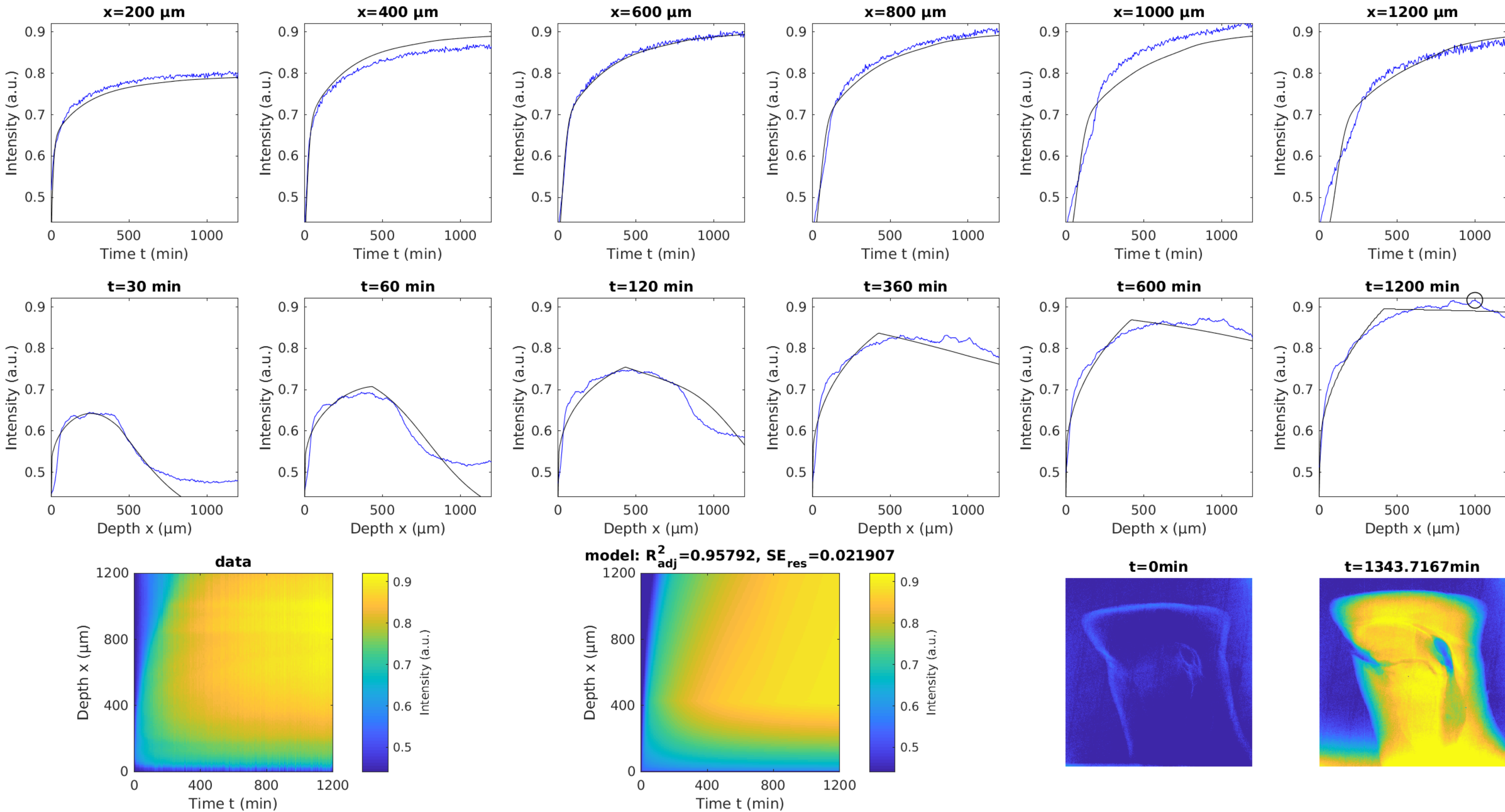

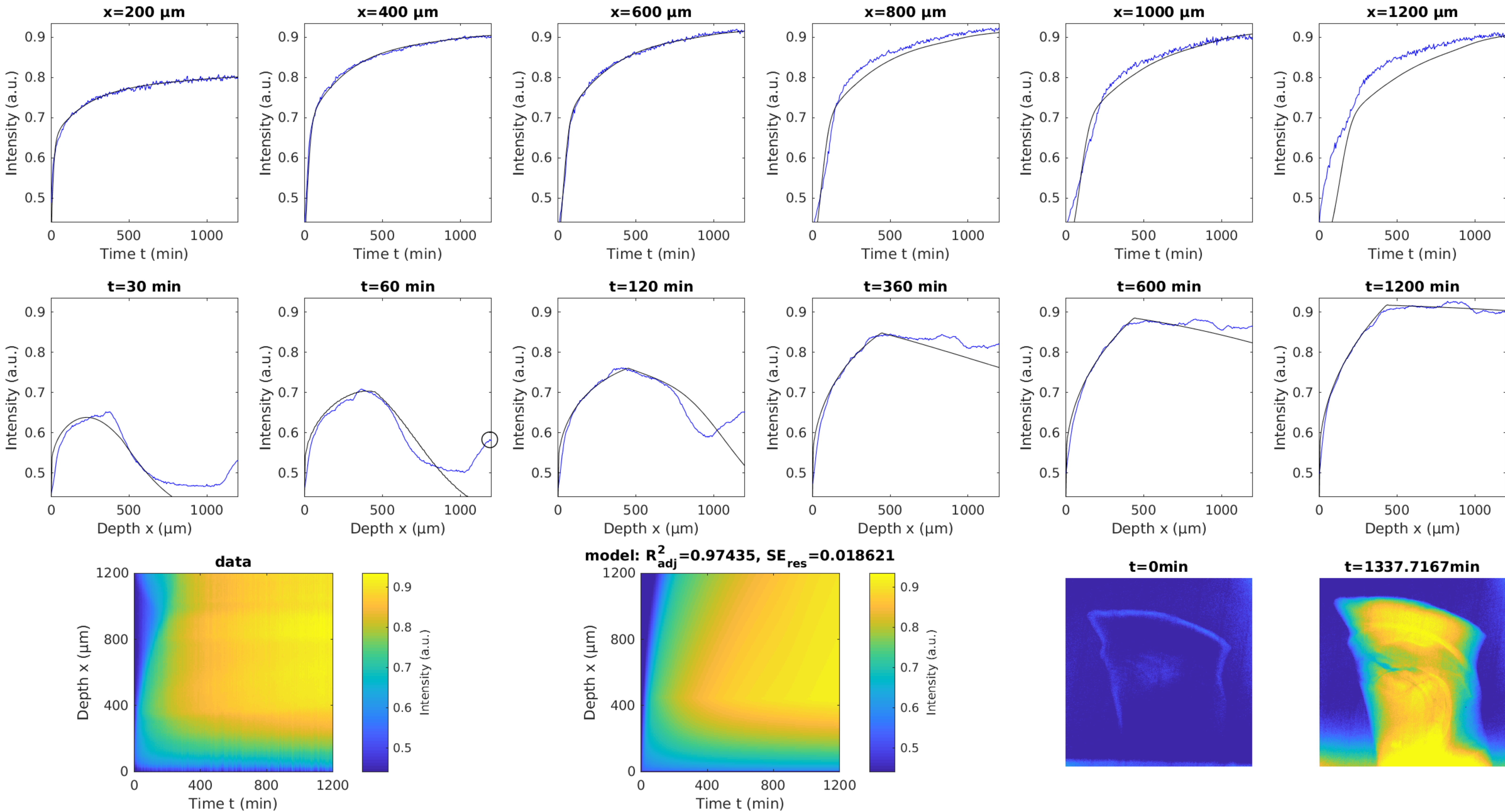

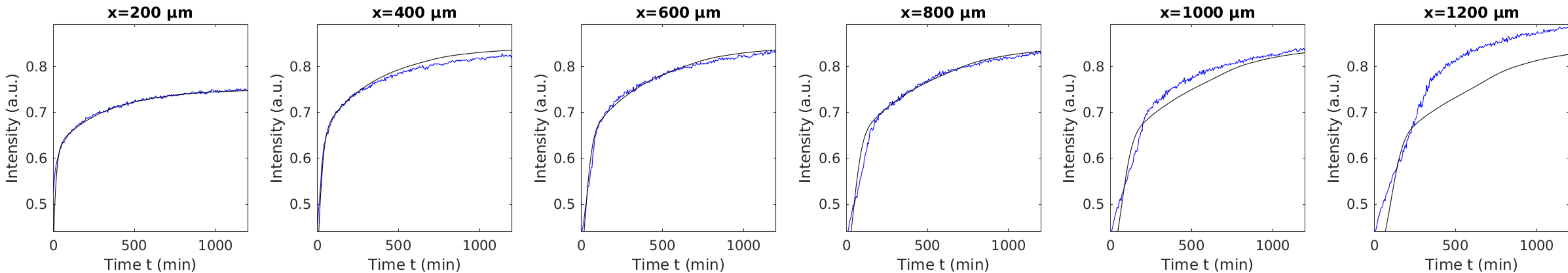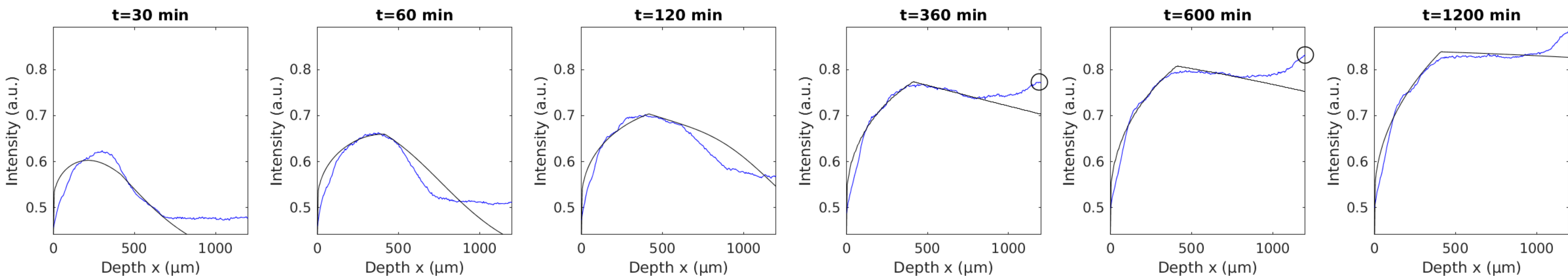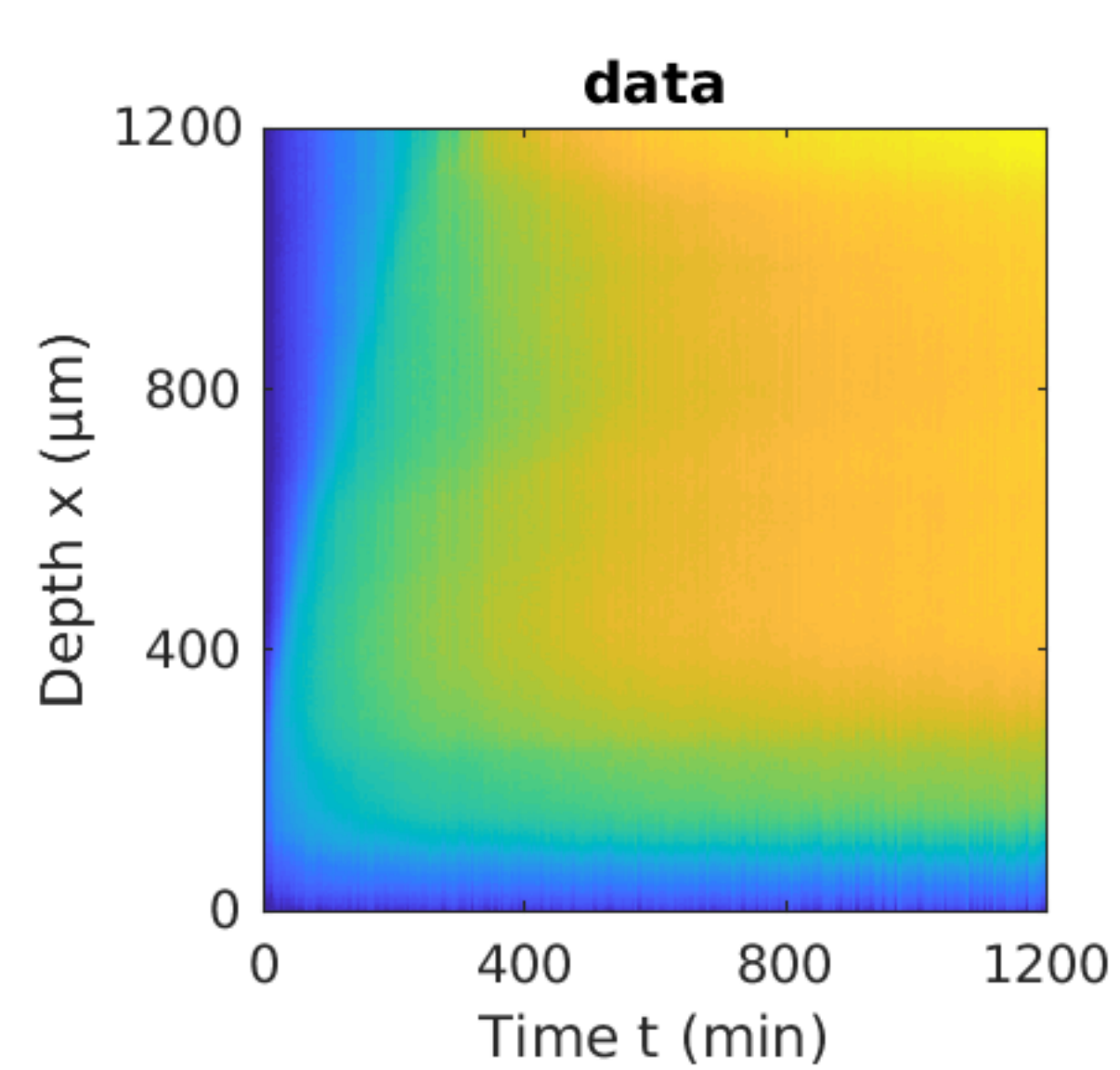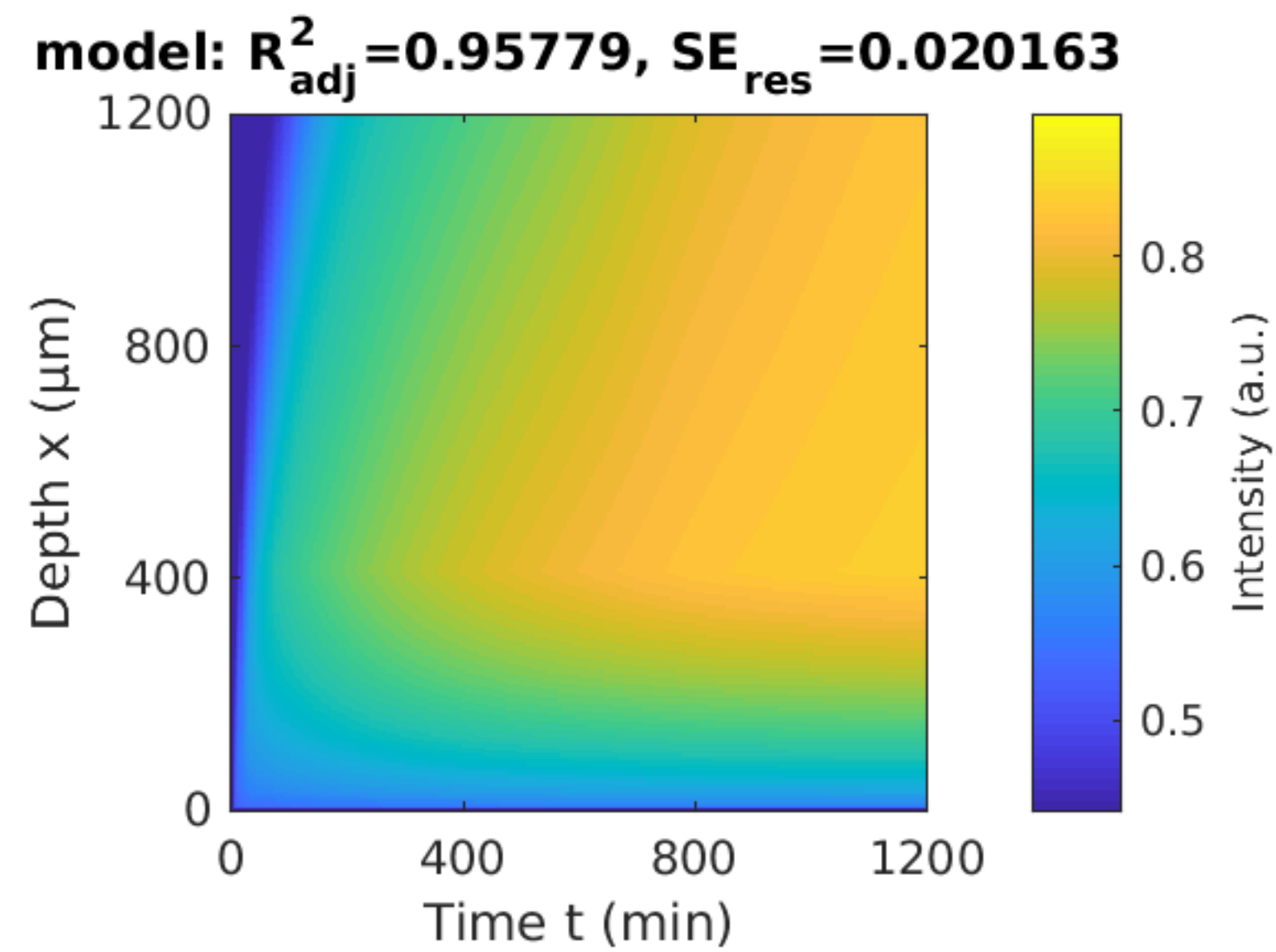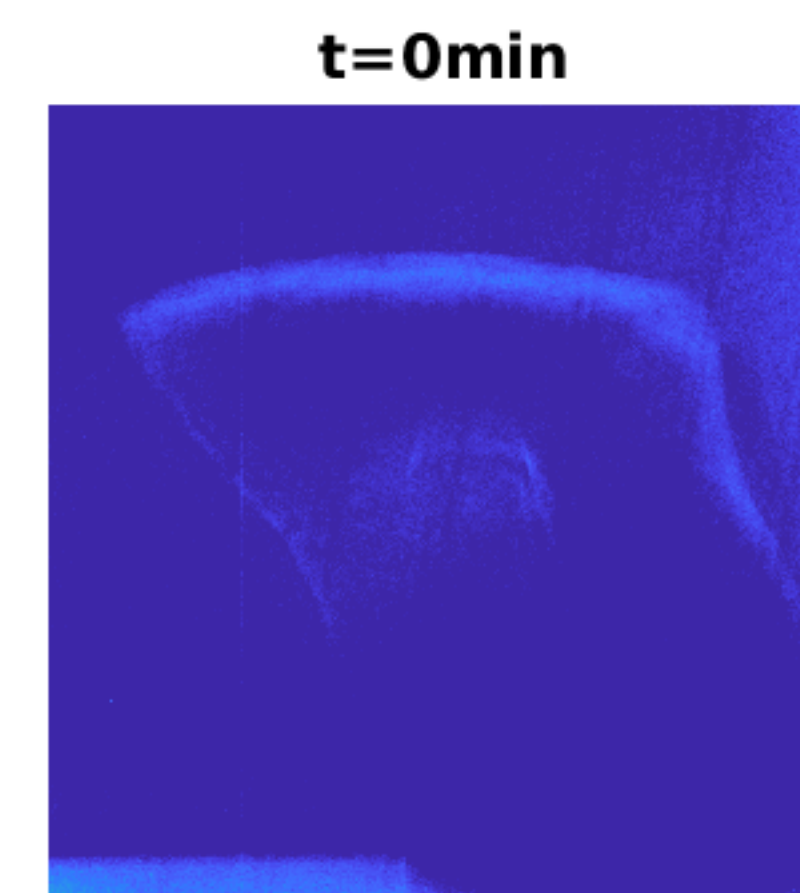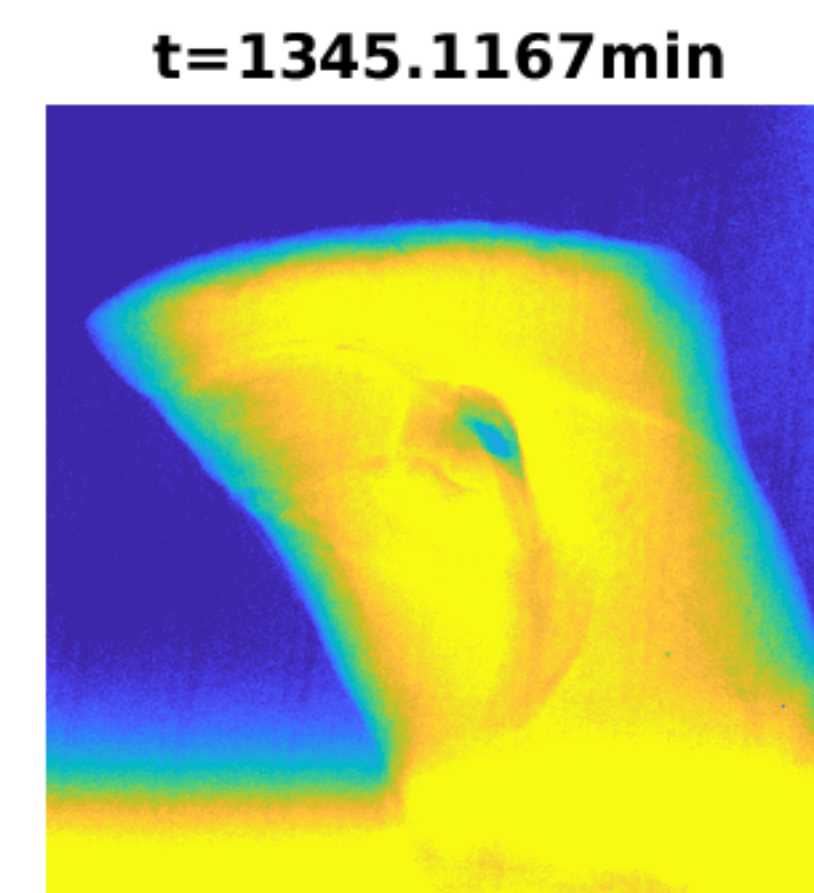

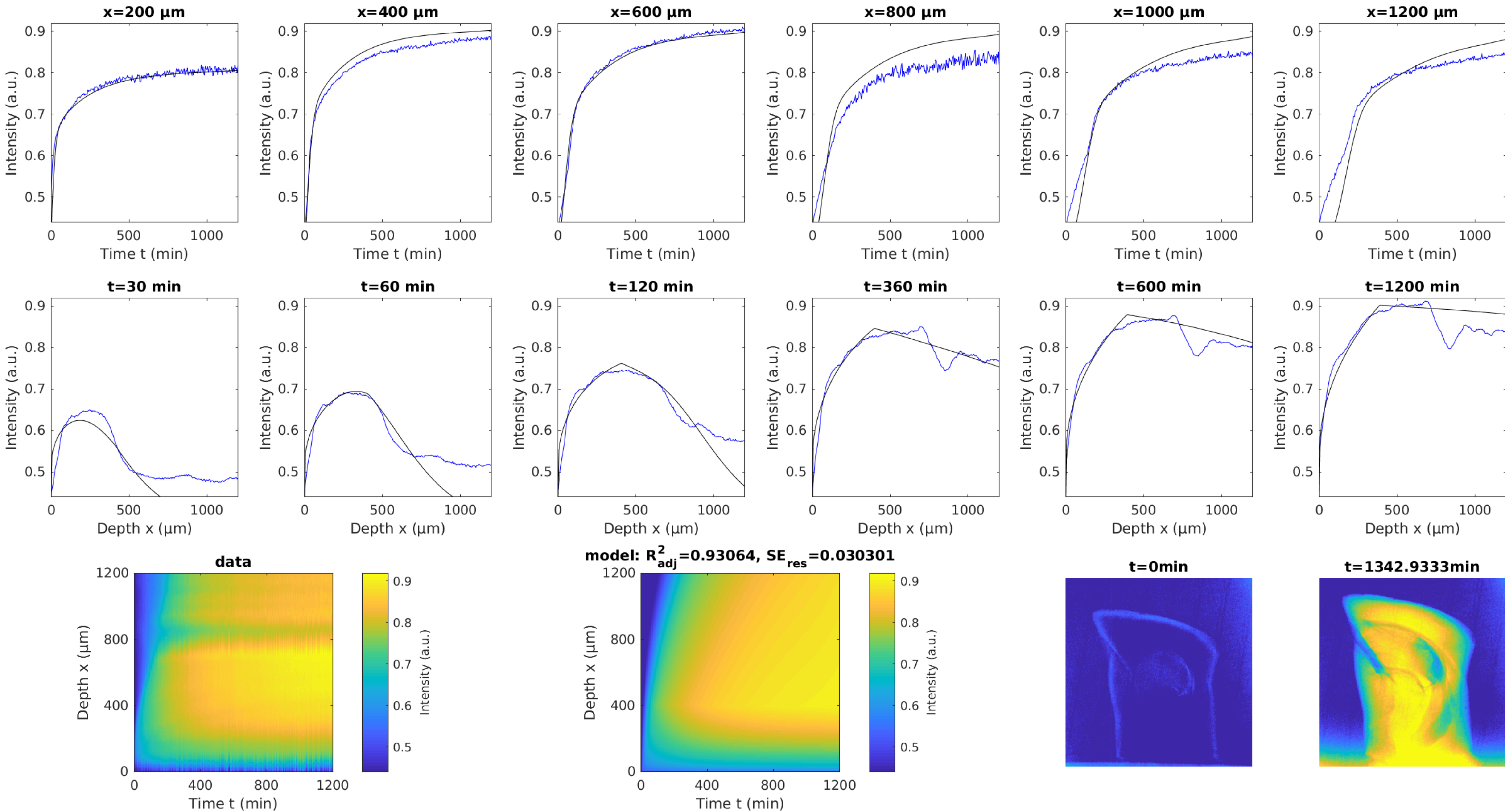

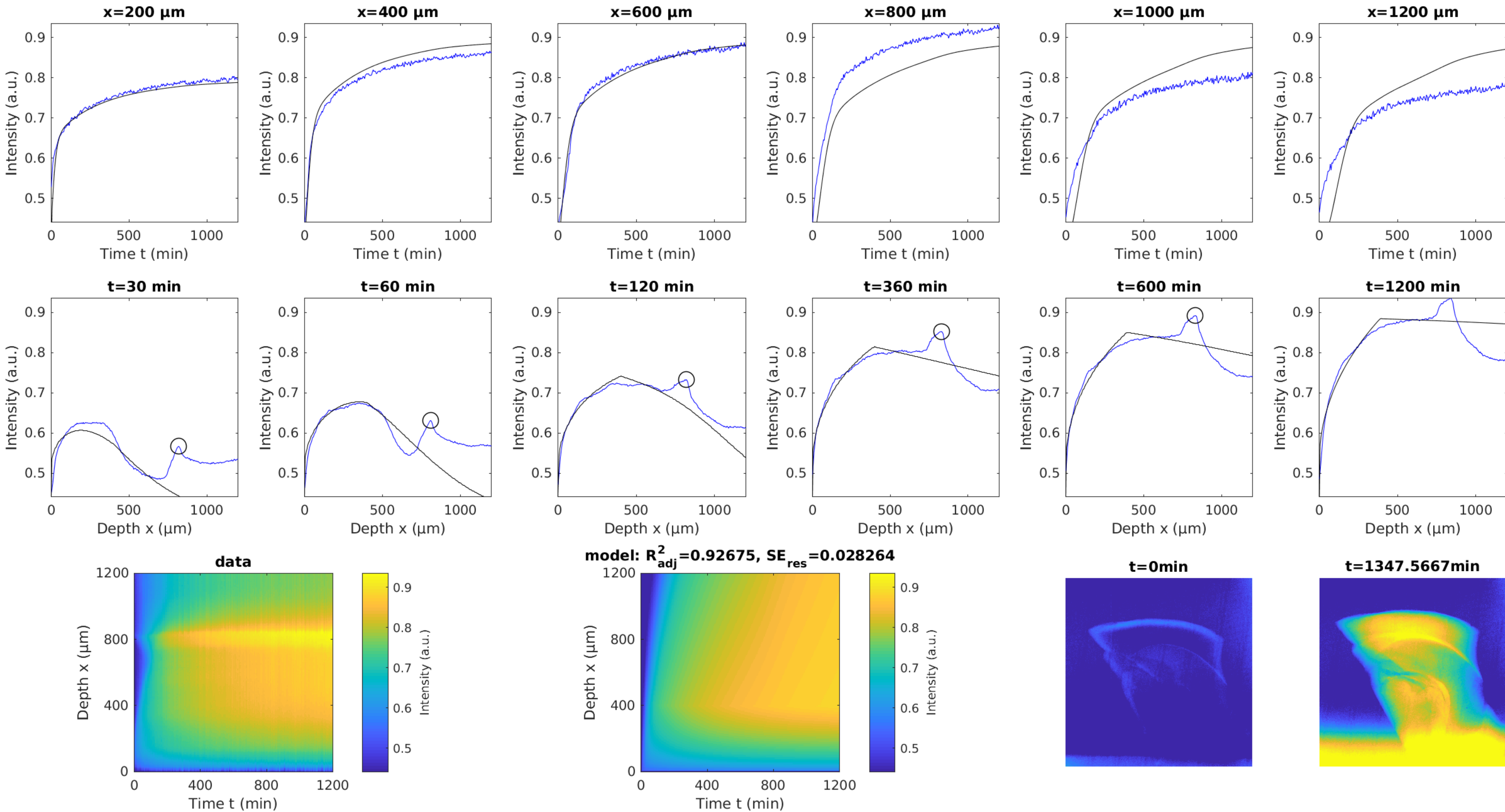

Supplement: Figure 1—source data 1. — The corresponding simulated model was fitted individually to the experimental data of each sample (black traces). The first row of every sample shows the temporal profile of osmium accumulation at different cortical depths, while the second row shows the spatial profile of osmium accumulation at different times after staining onset. The black circles indicate the location of axon tracts that tended to accumulate osmium faster and stronger. The third row shows the measured spatial-temporal accumulation of osmium (left), the corresponding fitted model simulations (middle) and the distribution of osmium in projection images of the sample at the beginning of the experiment and after about 22 hr of incubation (right). [file elife-72147-fig1-data1.pdf]

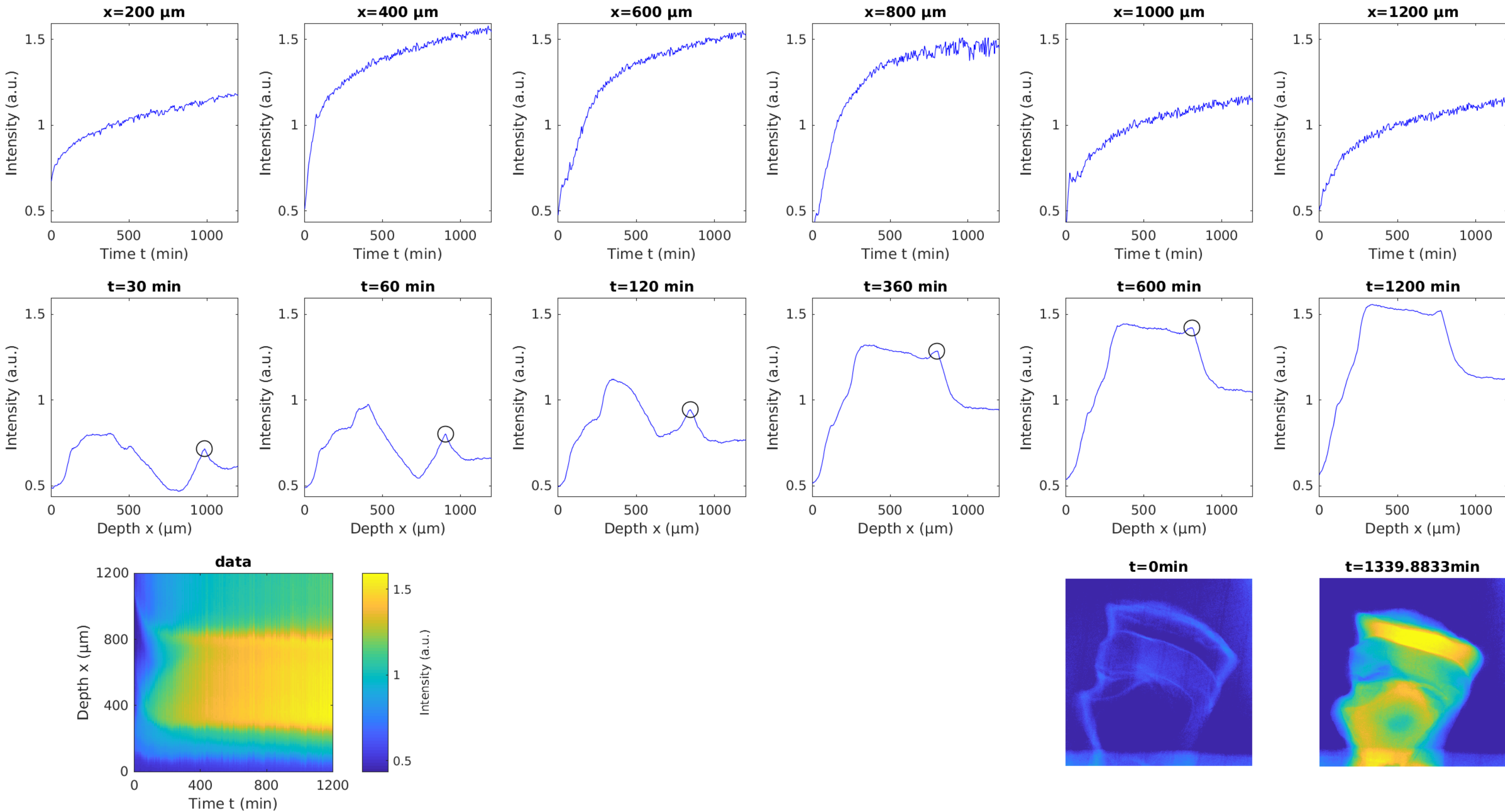

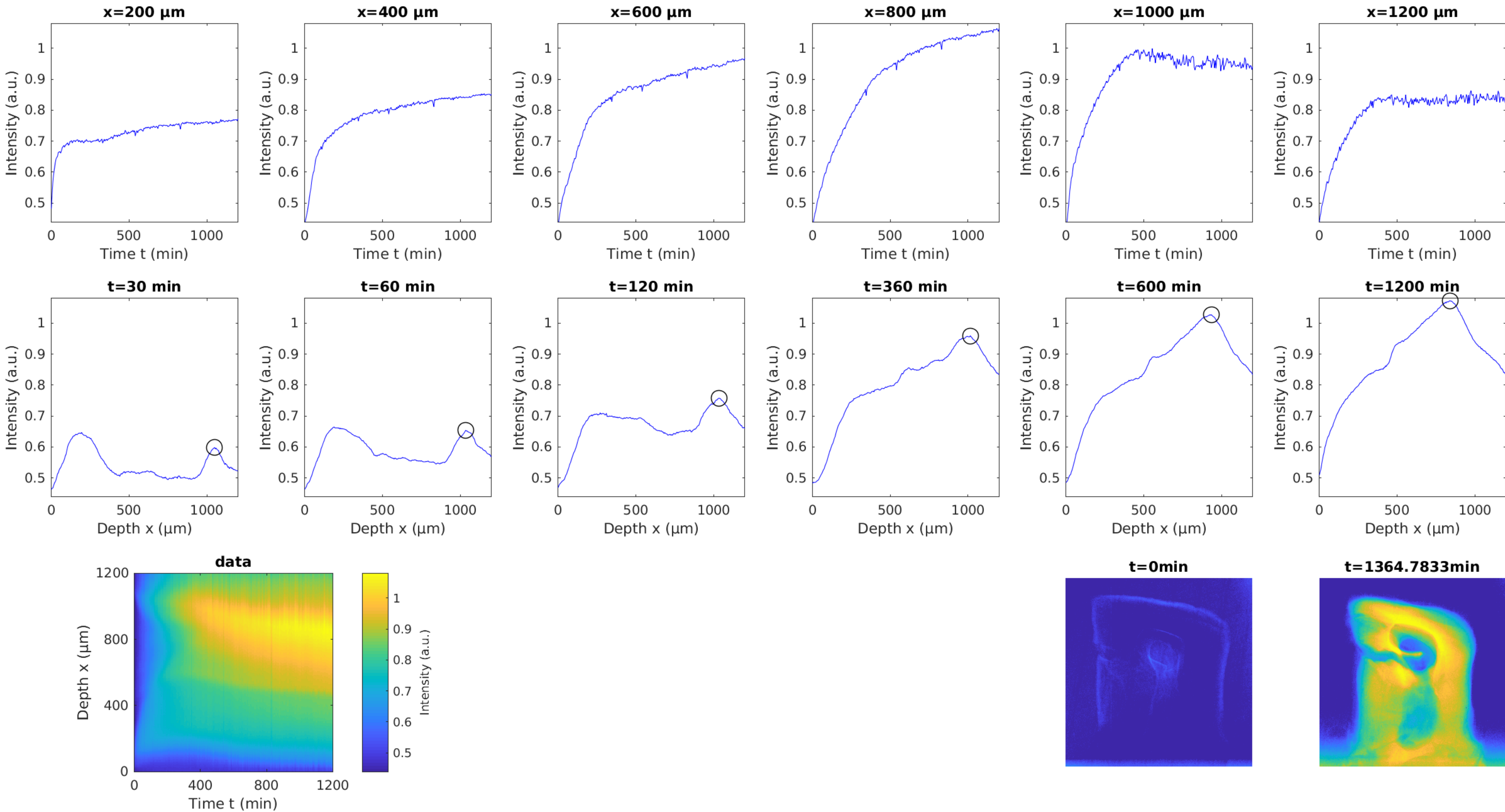

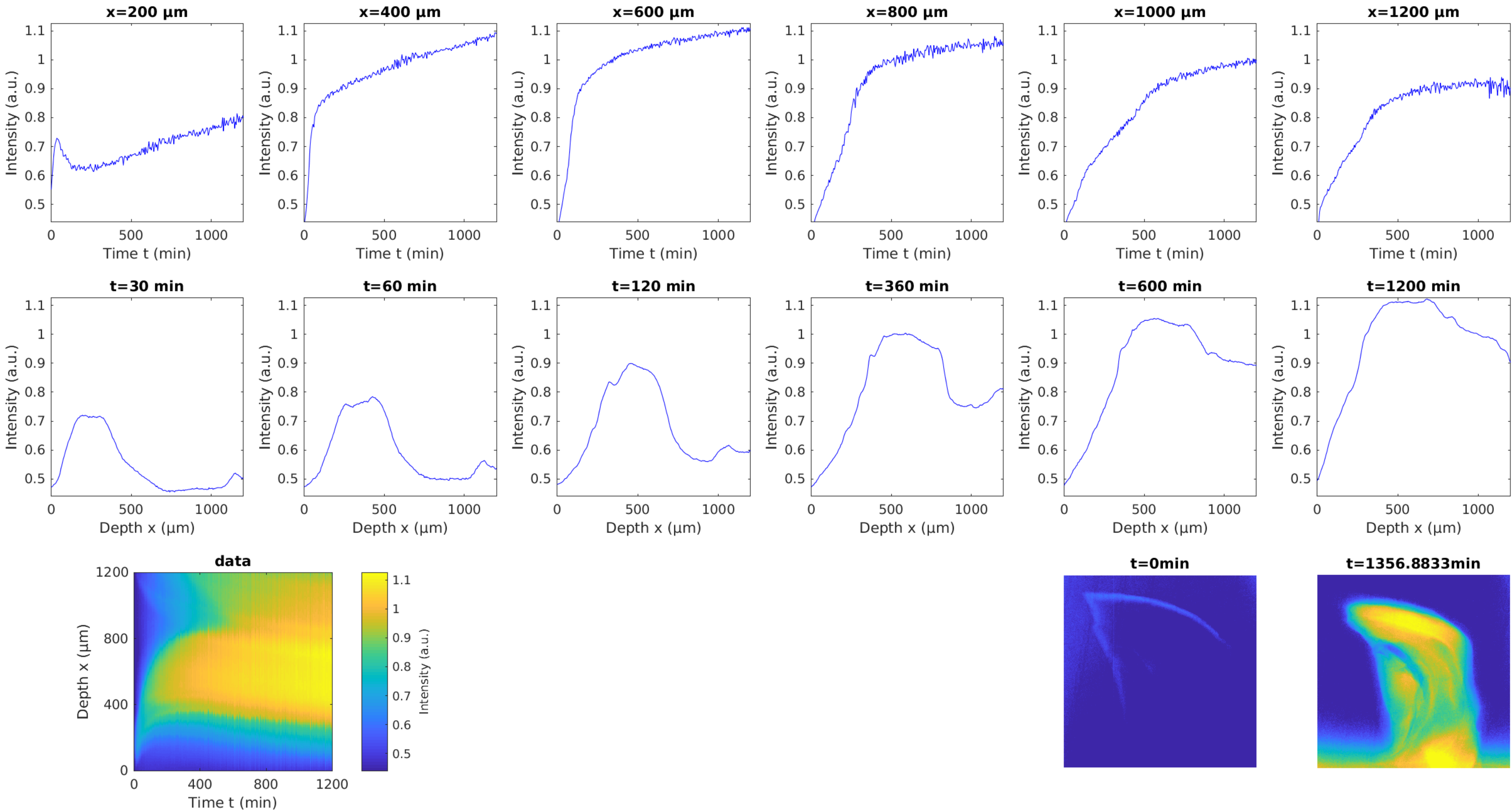

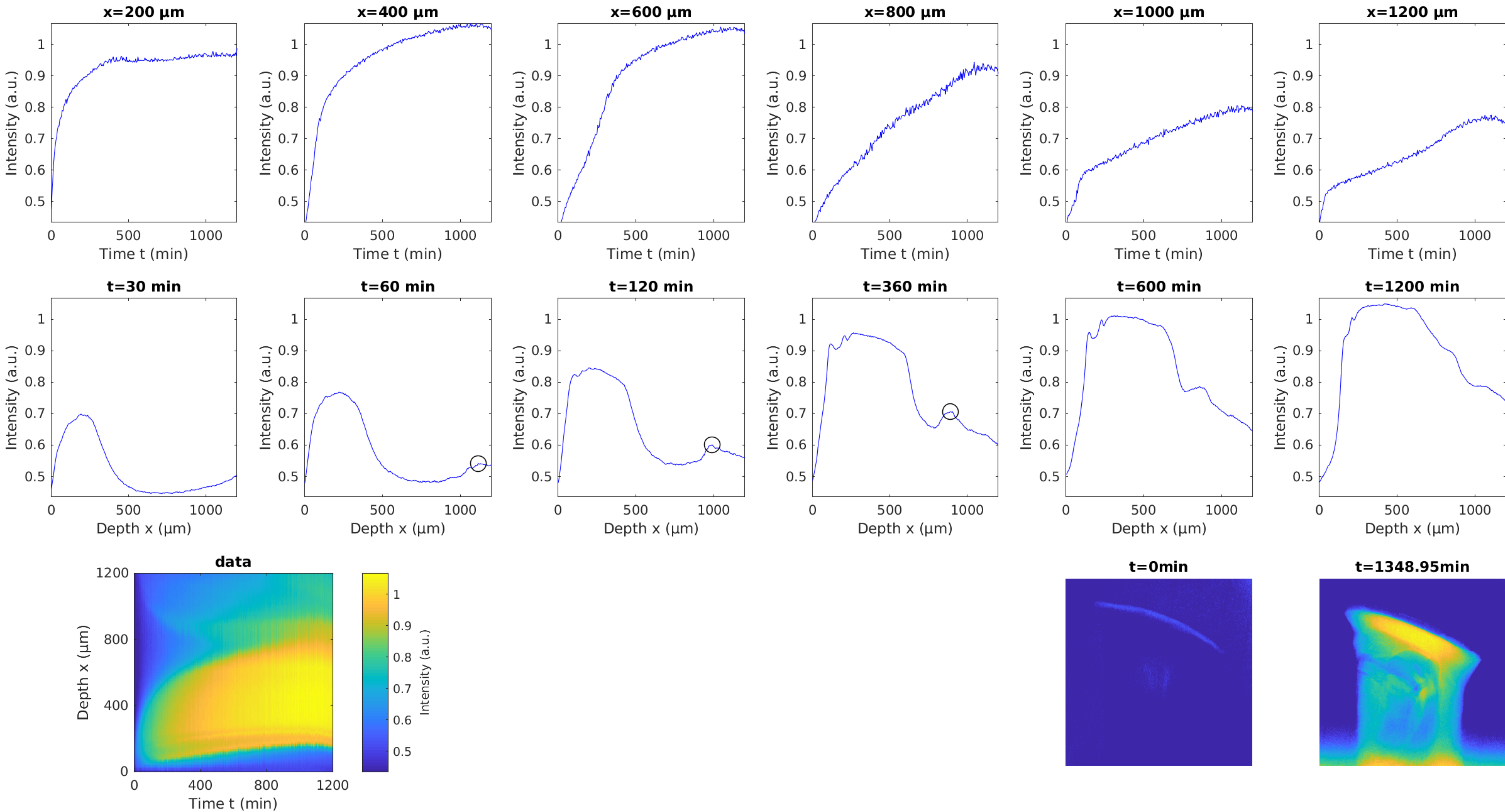

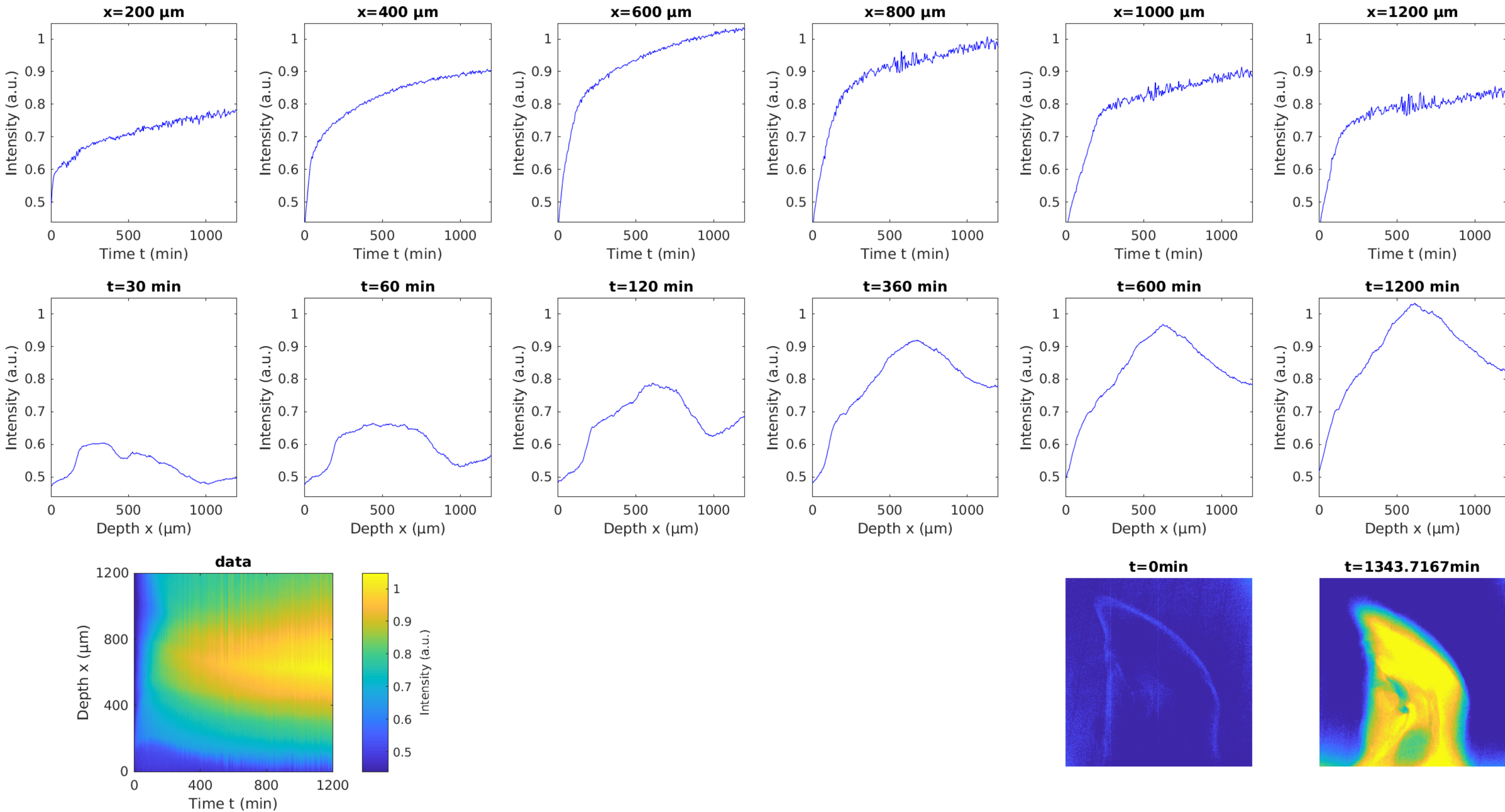

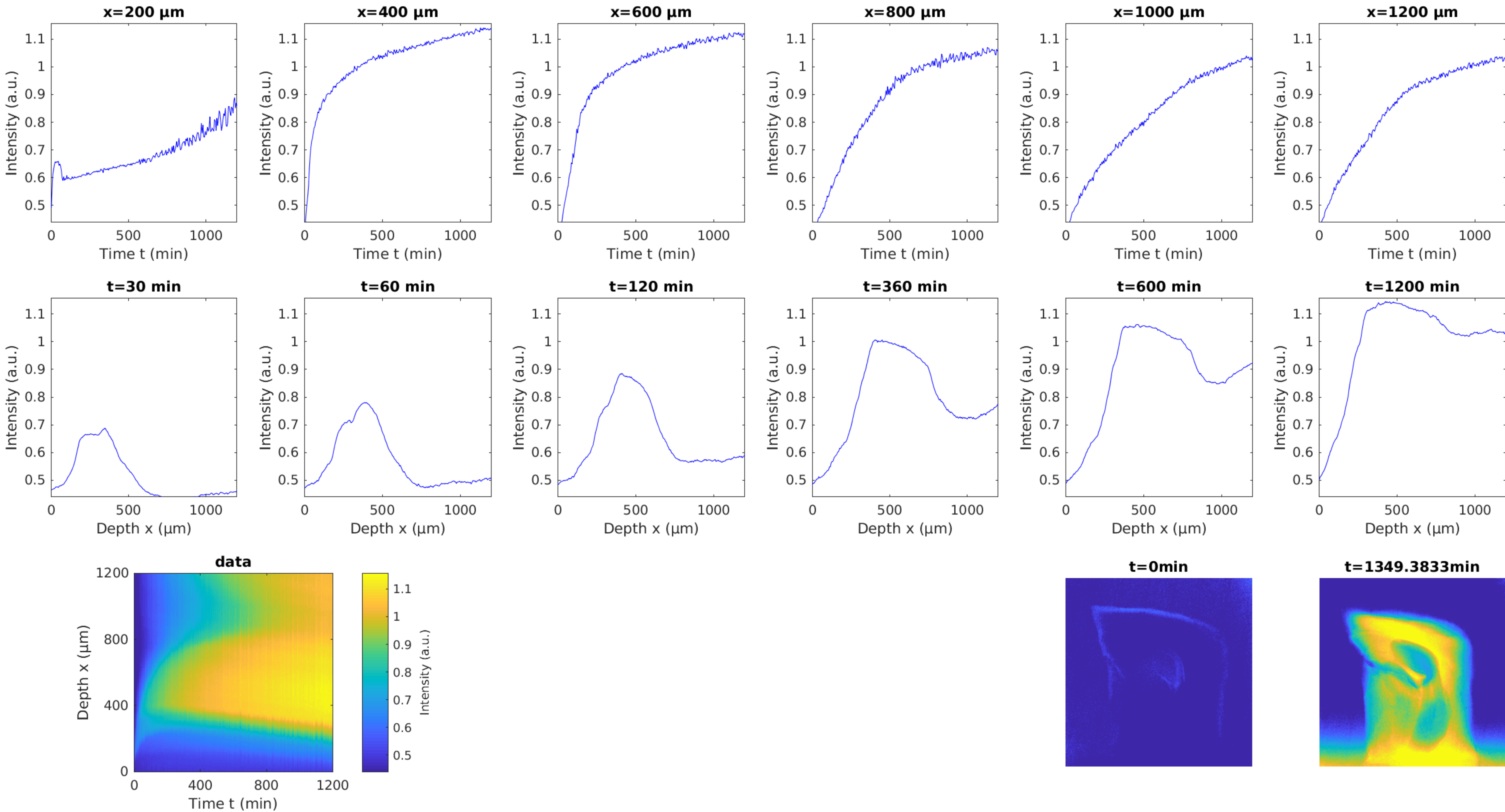

Supplement: Figure 1—source data 2. — The first row shows the temporal profile of osmium accumulation at different cortical depths, while the second row shows the spatial profile of osmium accumulation at different times after staining onset. The black circles indicate the location of axon tracts that tended to accumulate osmium faster and stronger. The third row shows the measured spatial-temporal accumulation of osmium (left) and the corresponding distribution of osmium in projection images of the sample at the beginning of the experiment and after about 22 hoursr of incubation (right). [file elife-72147-fig1-data2.pdf]

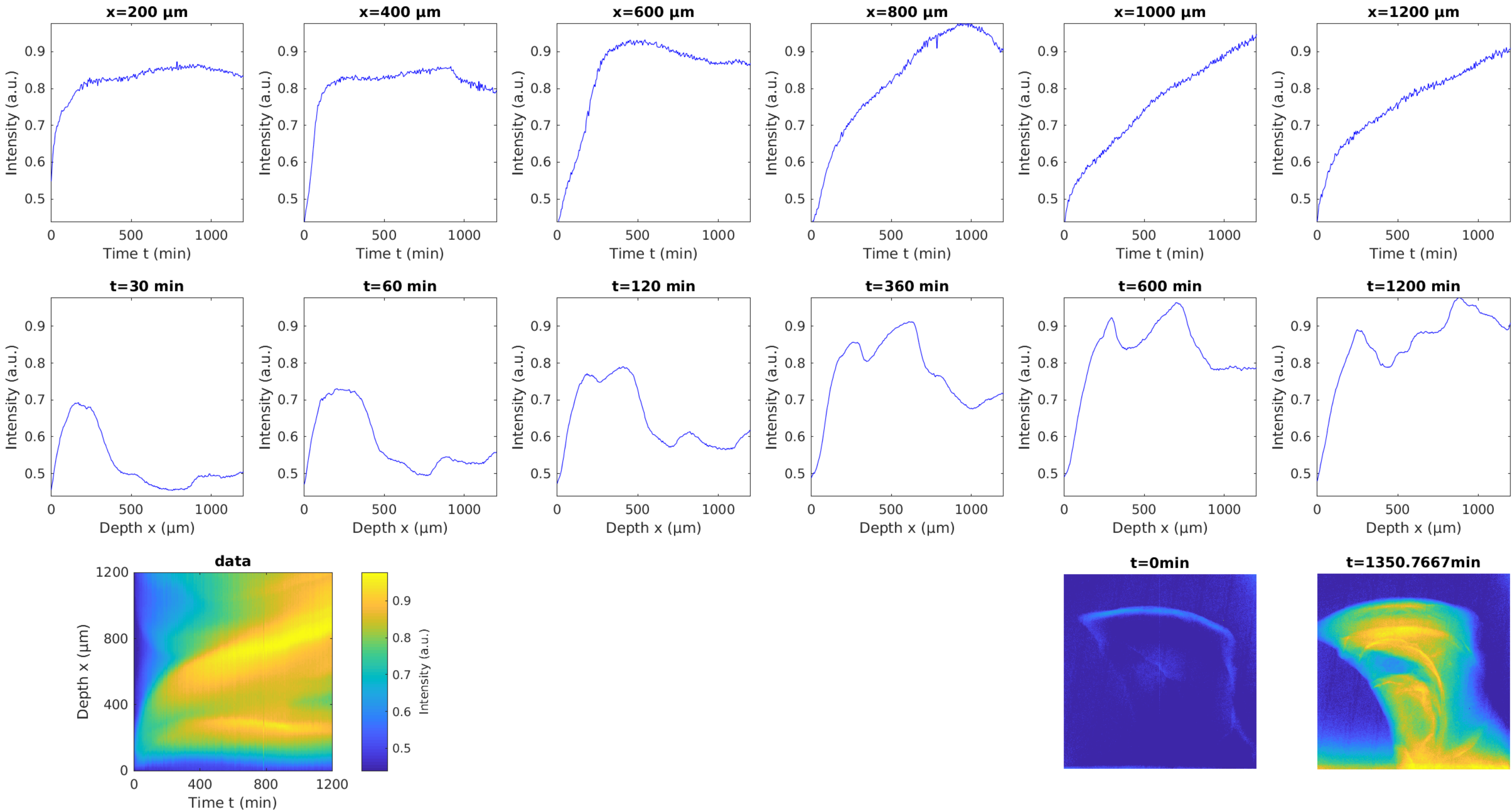

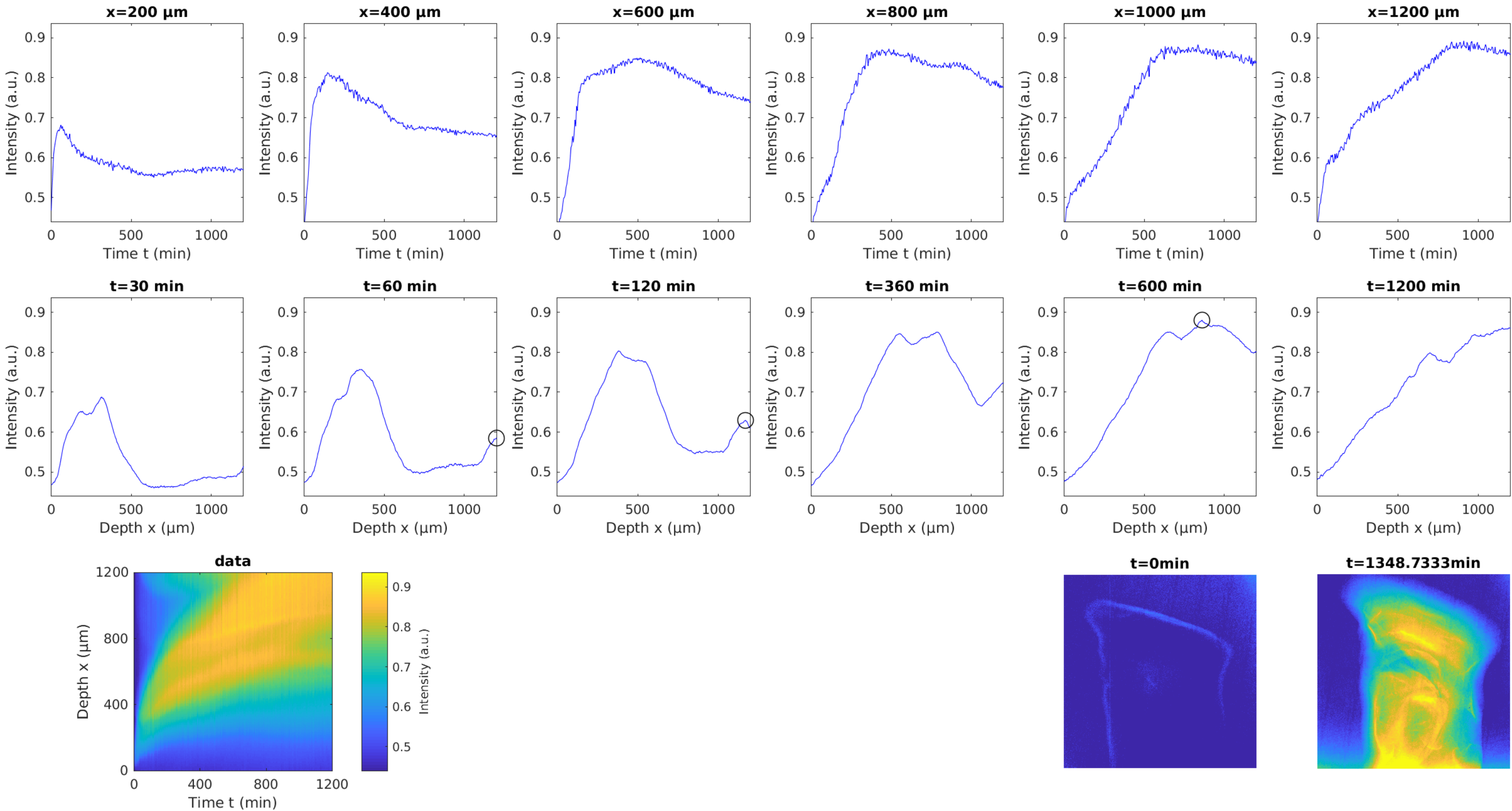

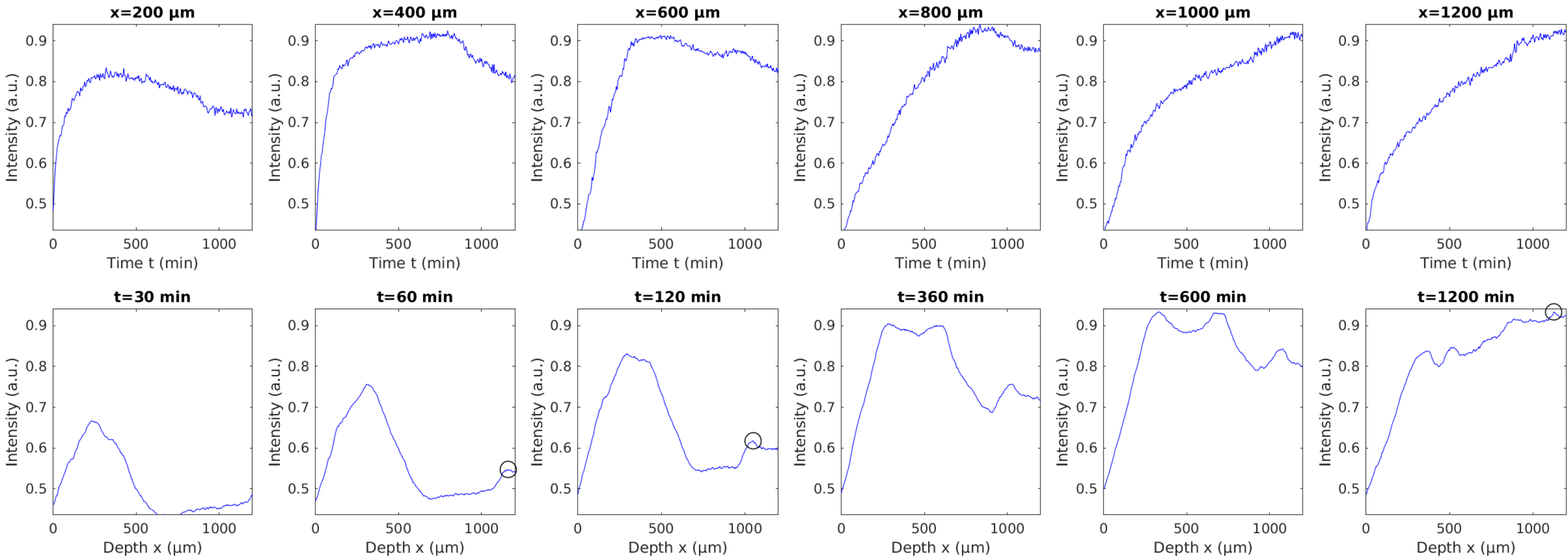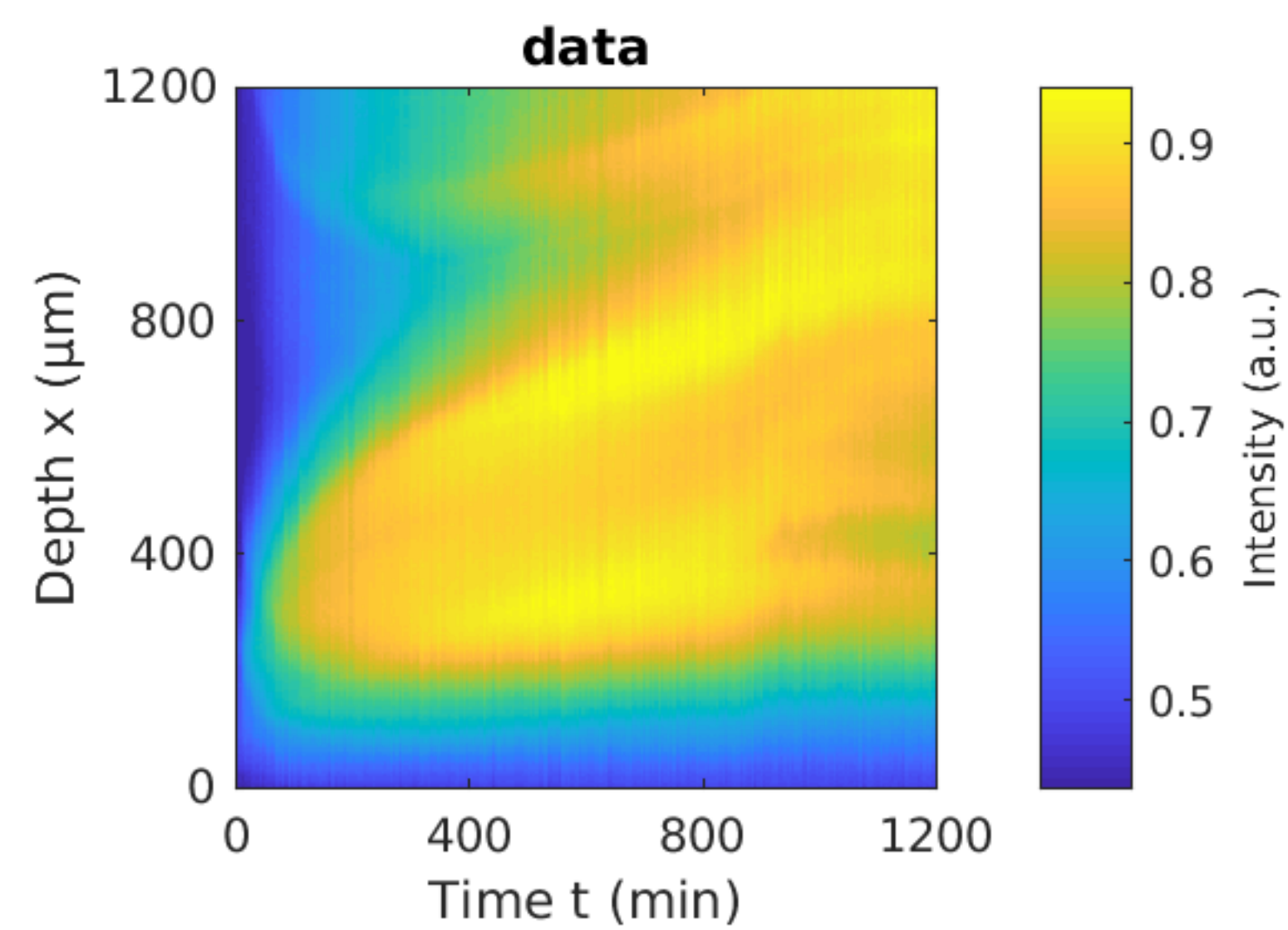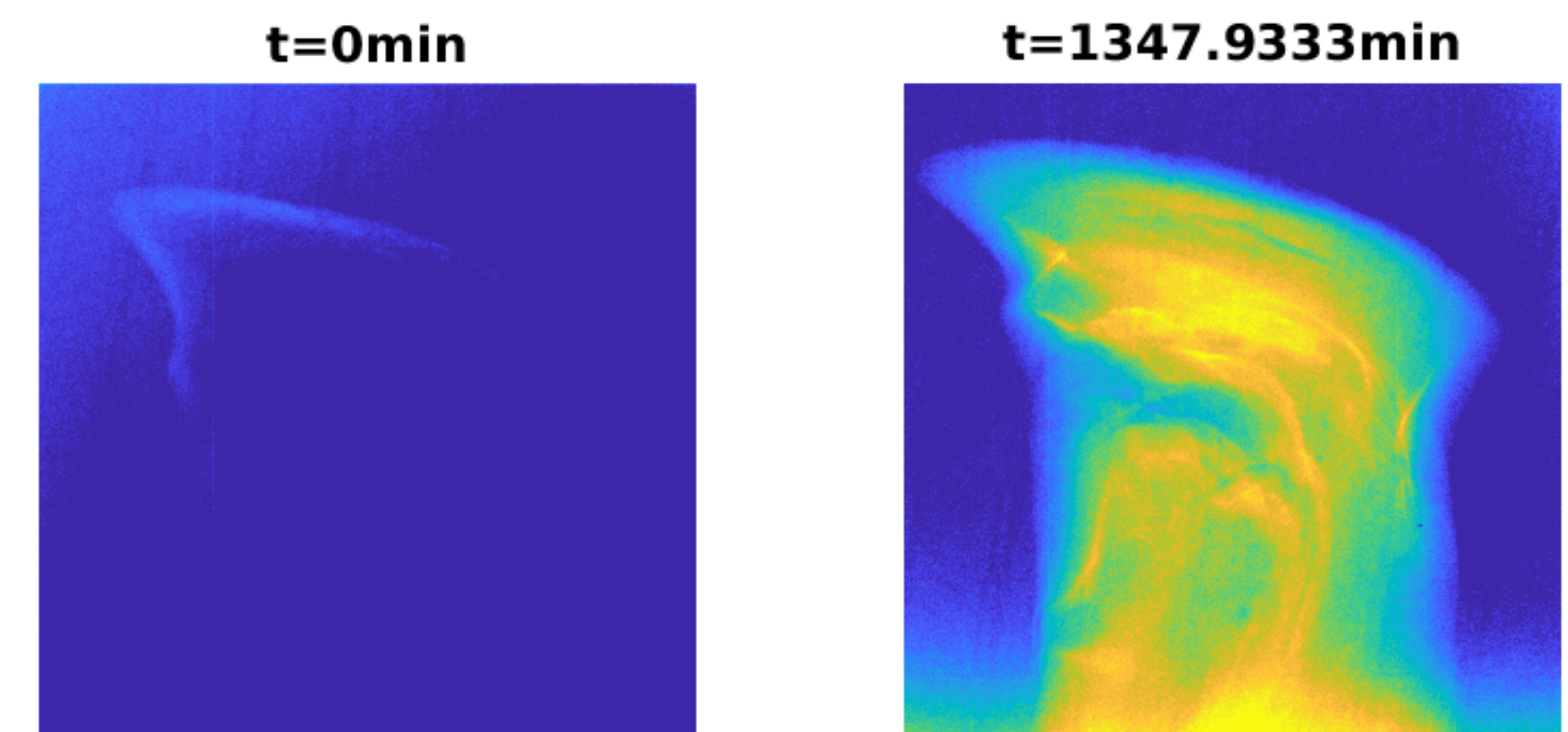

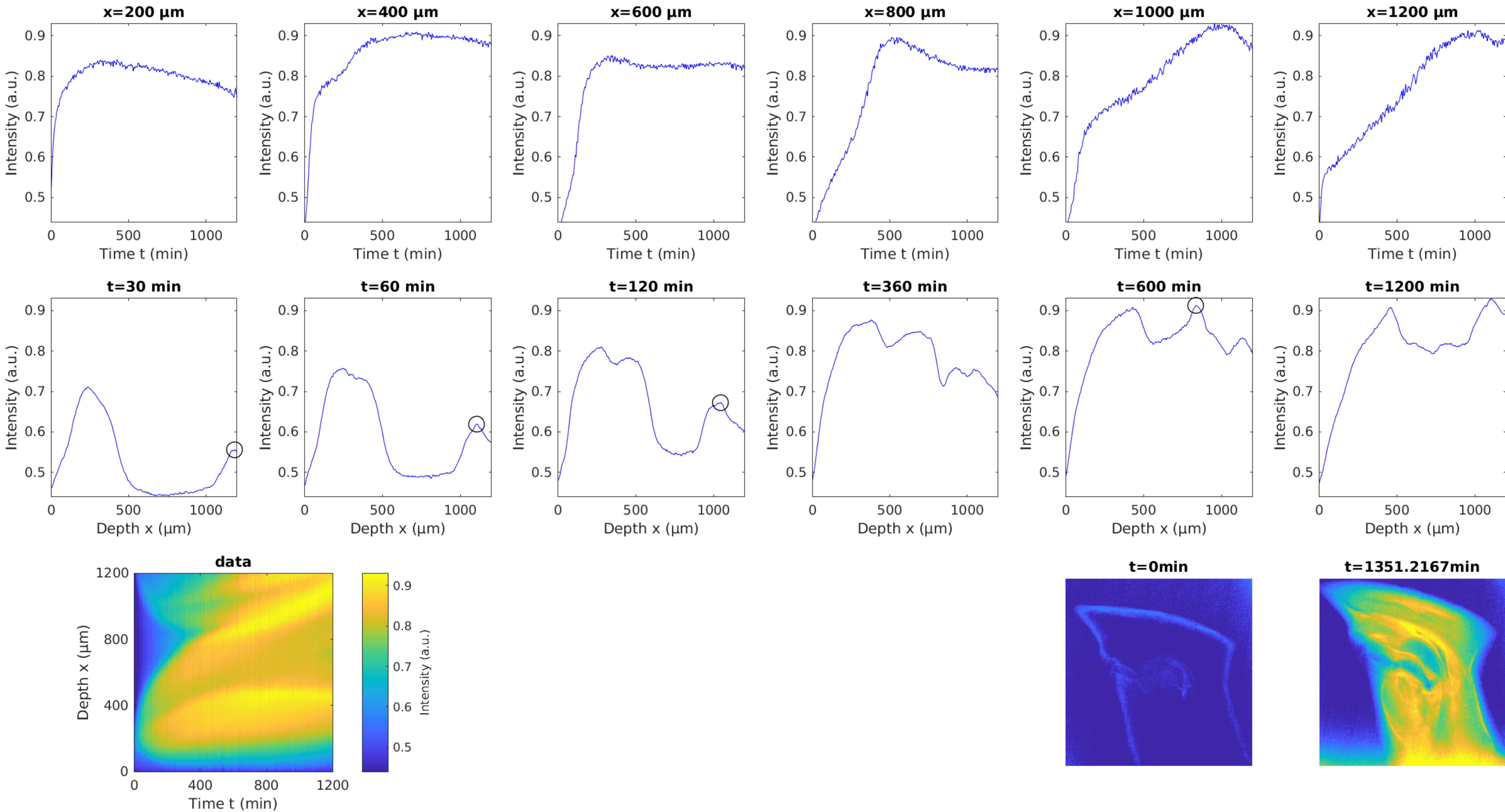

Supplement: Figure 1—source data 3. — The first row of every sample shows the temporal profile of osmium accumulation at different cortical depths, while the second row shows the spatial profile of osmium accumulation at different times after staining onset. The black circles indicate the location of axon tracts that tended to accumulate osmium faster and stronger. The third row shows the measured spatial-temporal accumulation of osmium (left) and the corresponding distribution of osmium in projection images of the sample at the beginning of the experiment and after about 22 hoursr of incubation (right). [file elife-72147-fig1-data3.pdf]

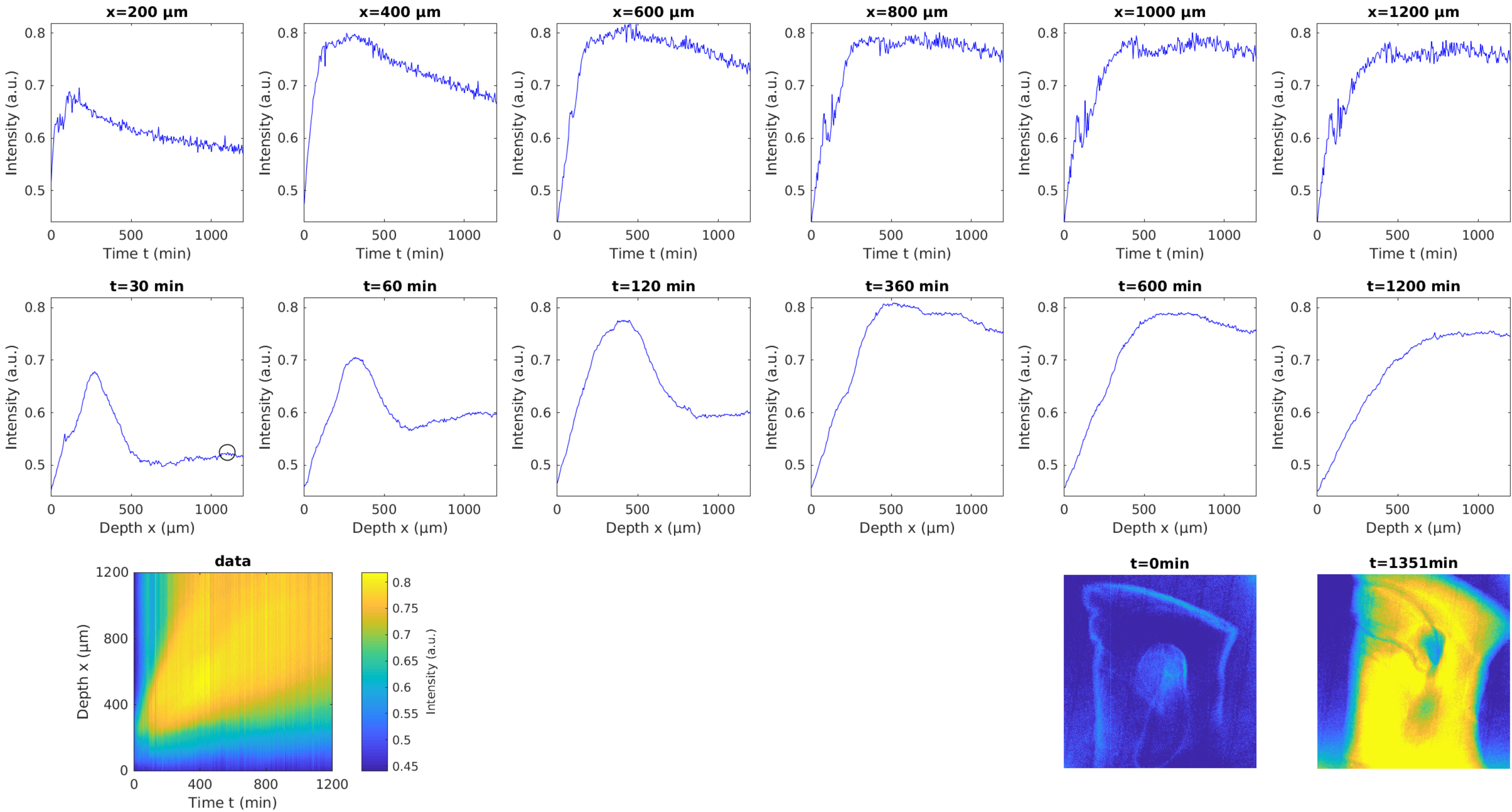

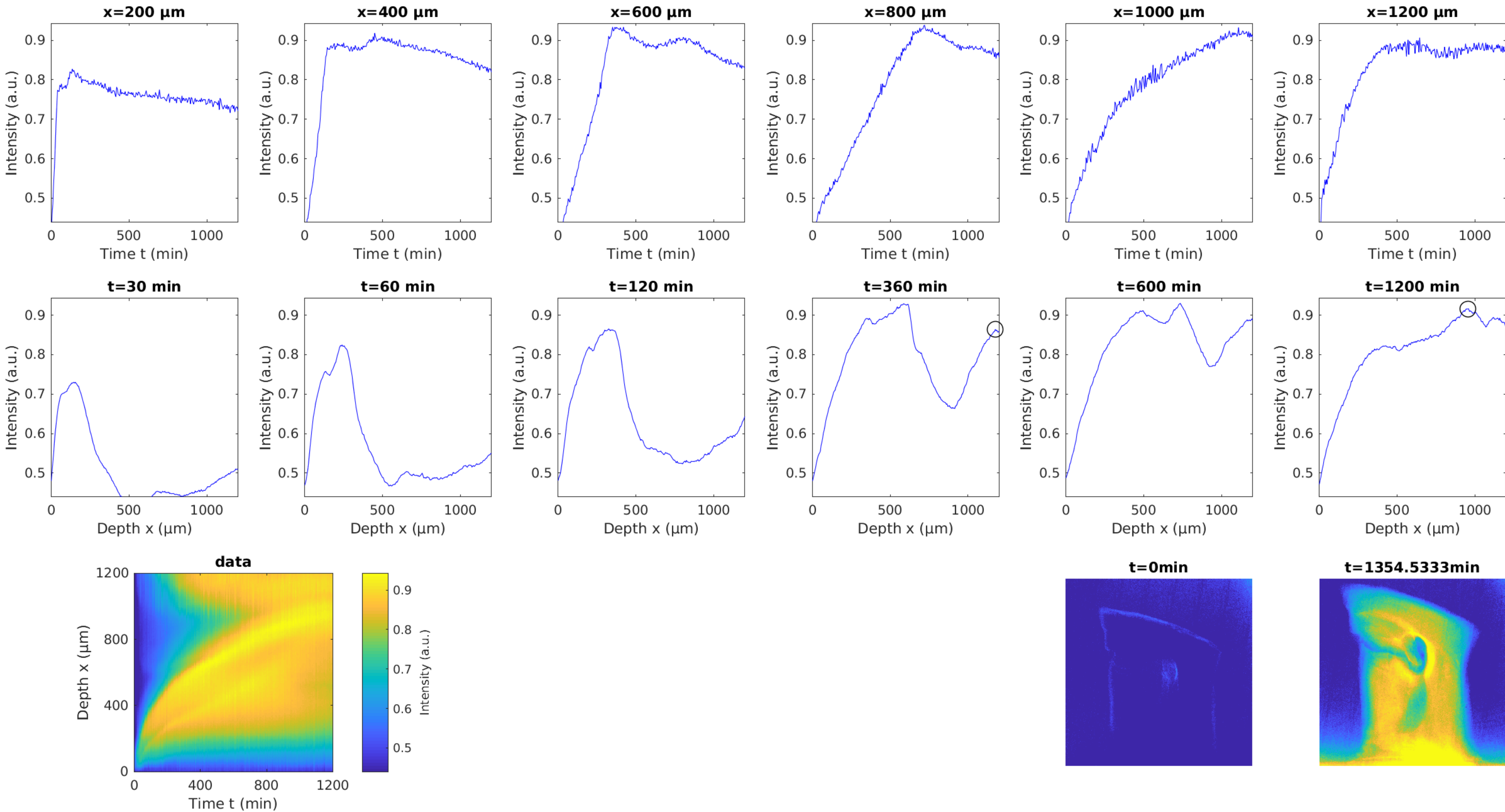

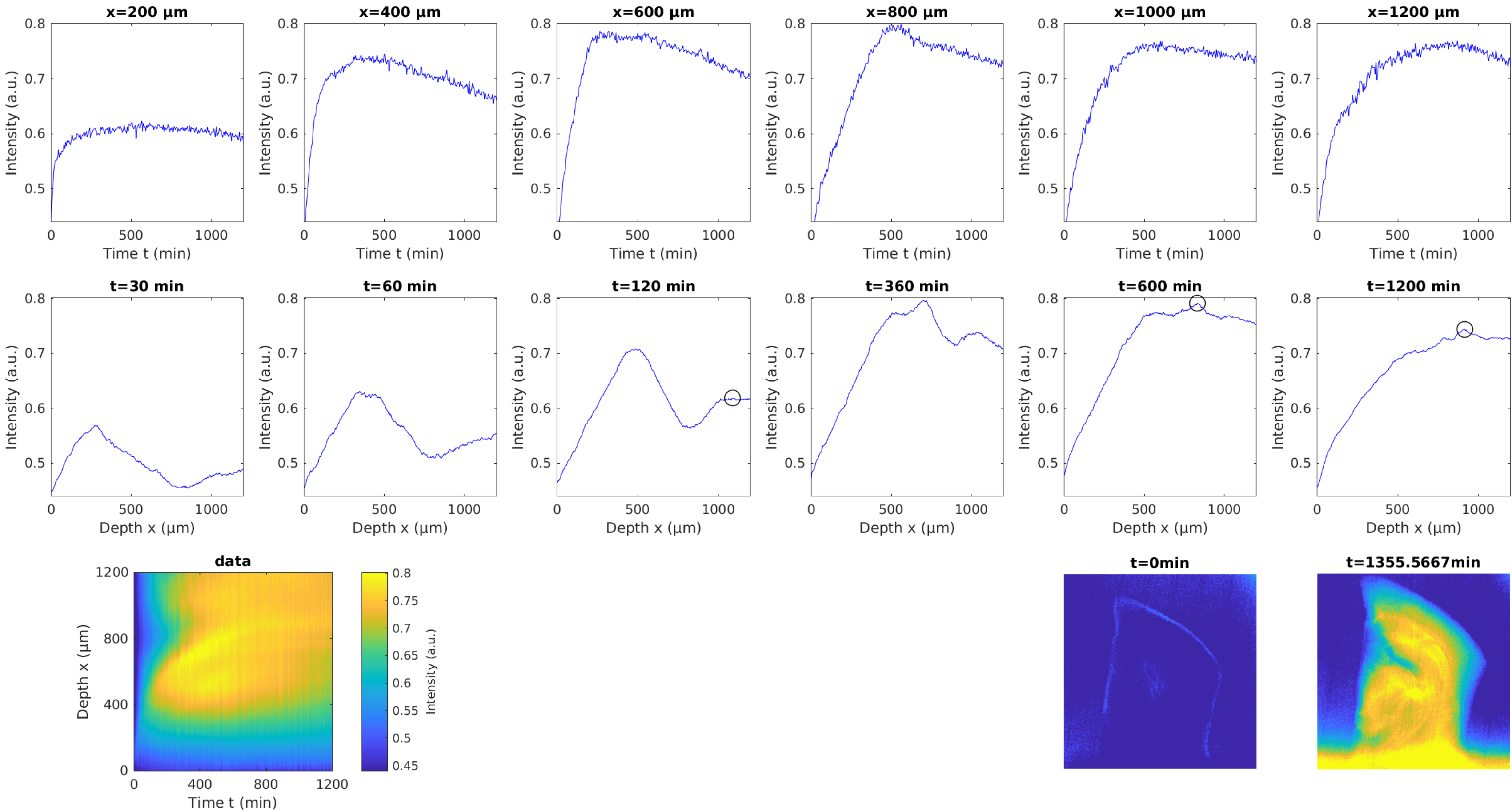

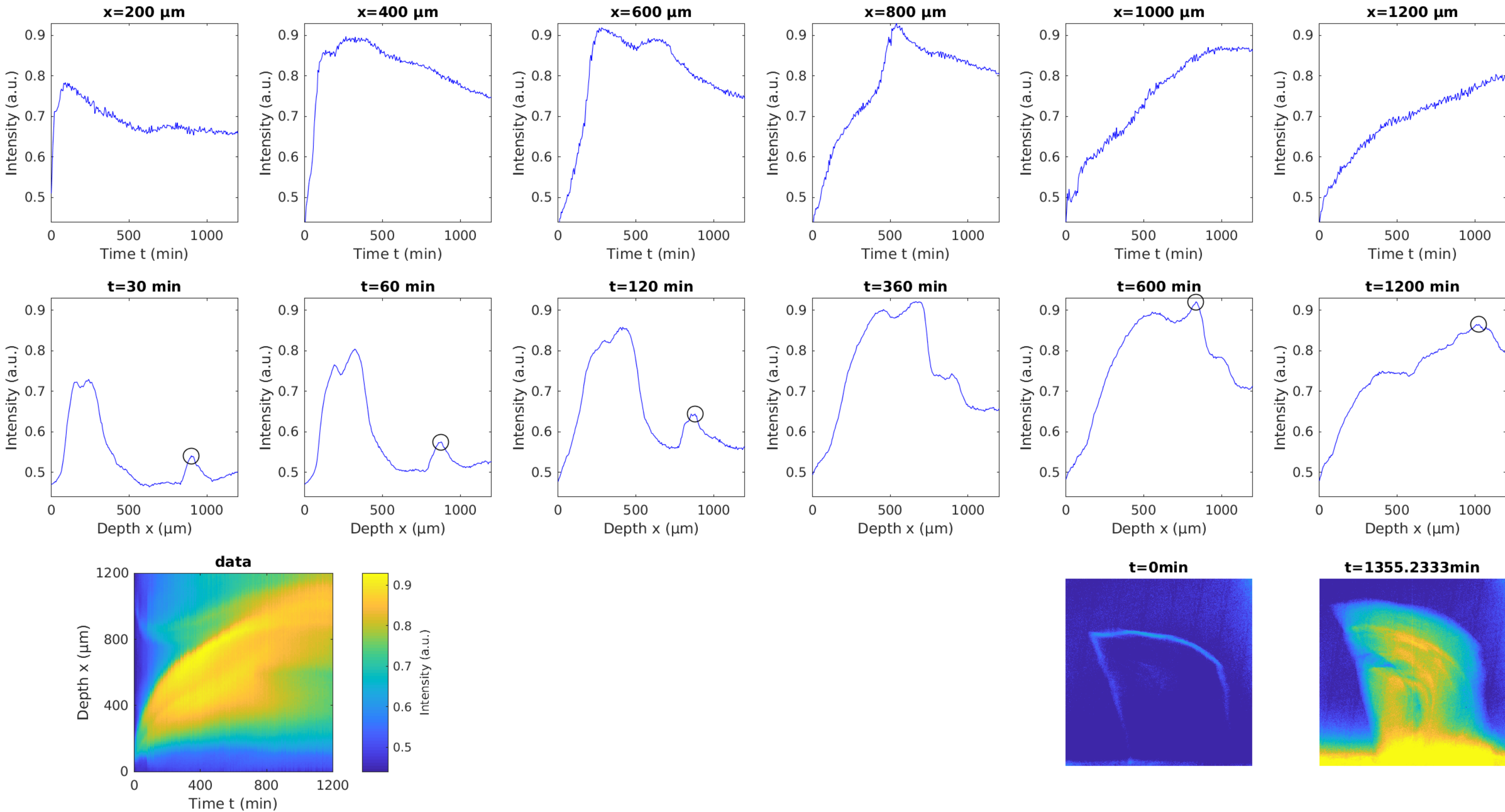

Supplement: Figure 1—source data 4. — The first row of every sample shows the temporal profile of osmium accumulation at different cortical depths, while the second row shows the spatial profile of osmium accumulation at different times after staining onset. The black circles indicate the location of axon tracts that tended to accumulate osmium faster and stronger. The third row shows the measured spatial-temporal accumulation of osmium (left) and the corresponding distribution of osmium in projection images of the sample at the beginning of the experiment and after about 22 hoursr of incubation (right). [file elife-72147-fig1-data4.pdf]

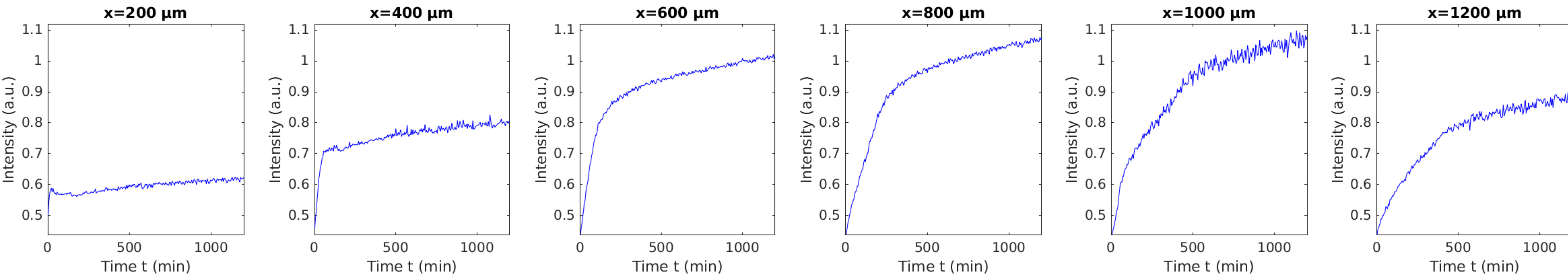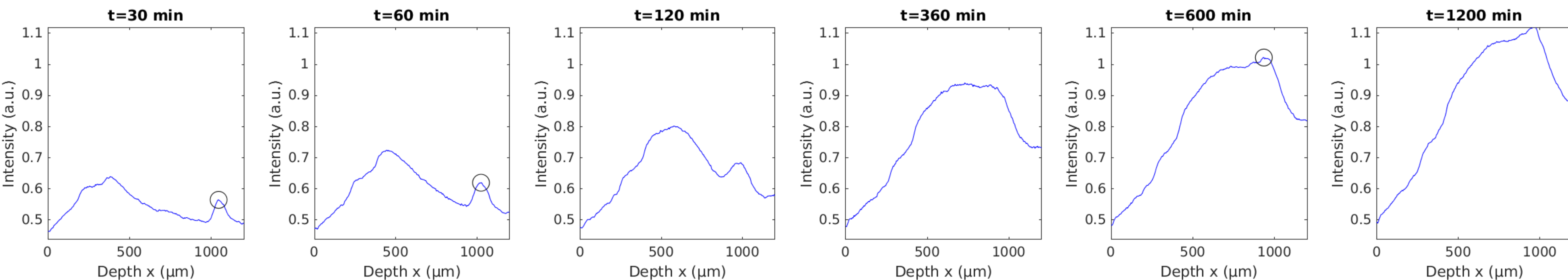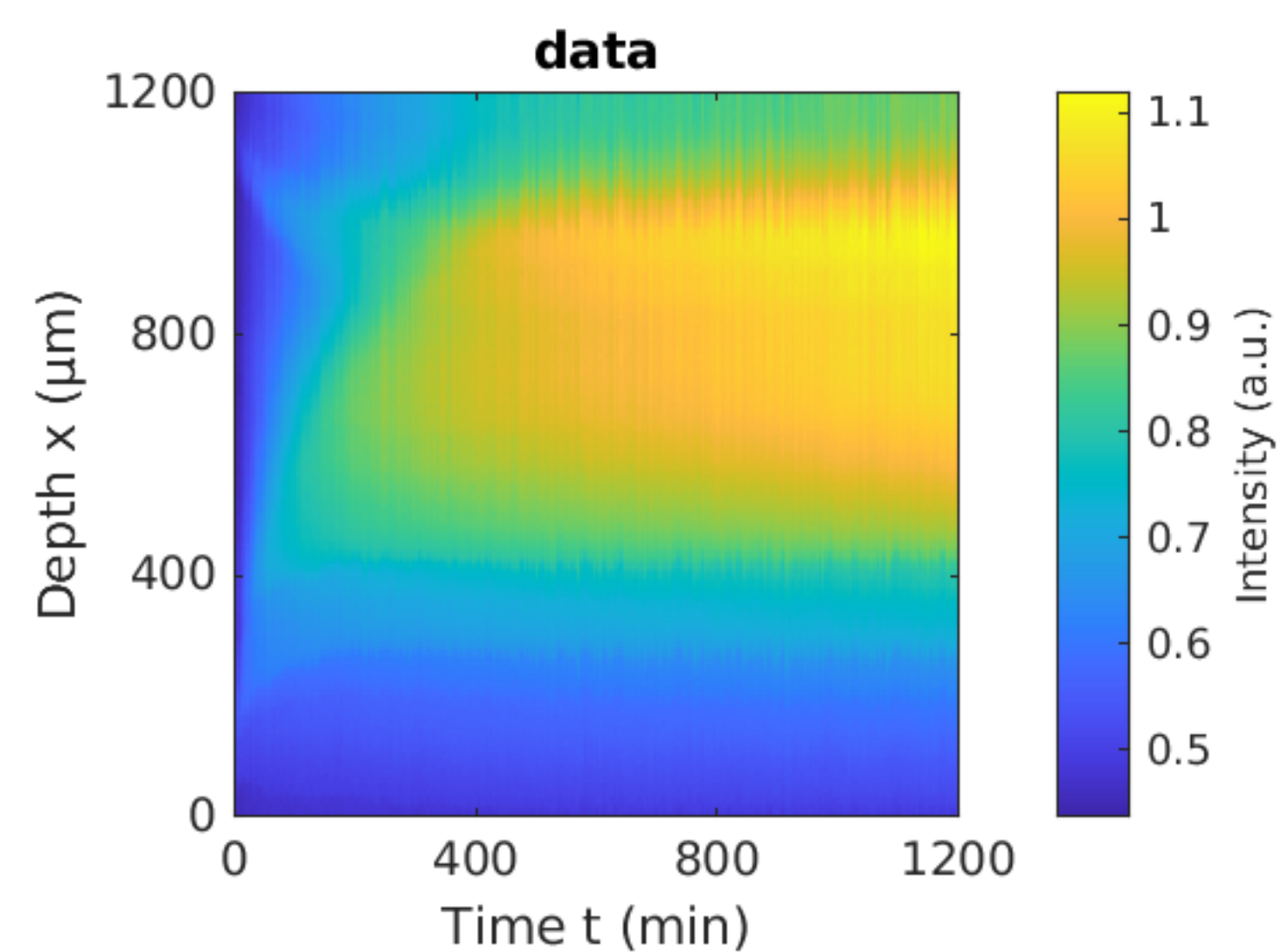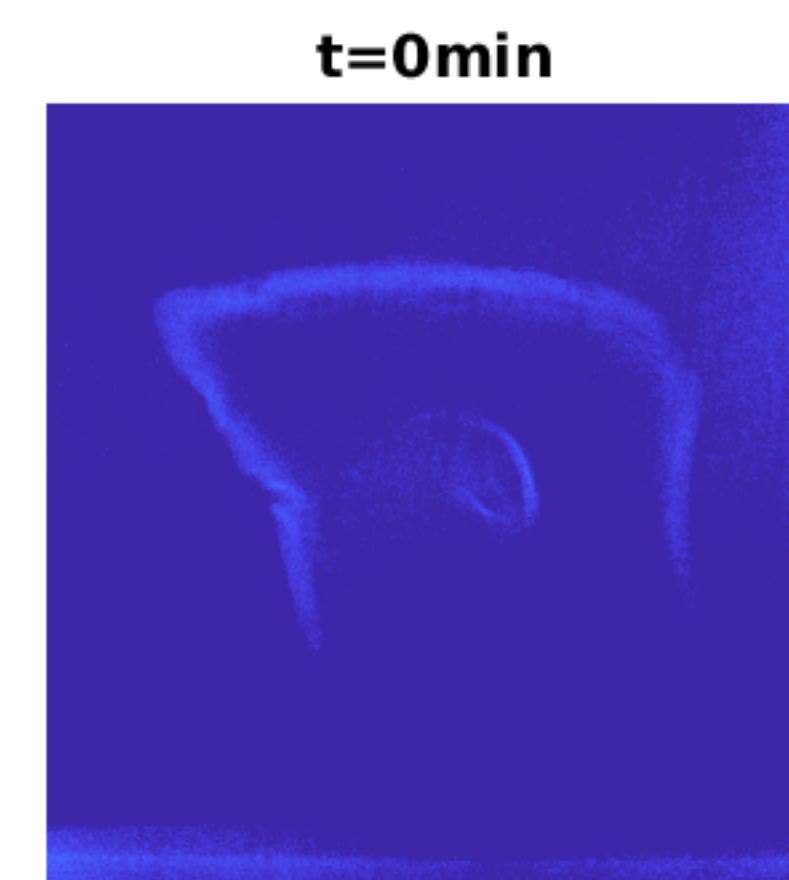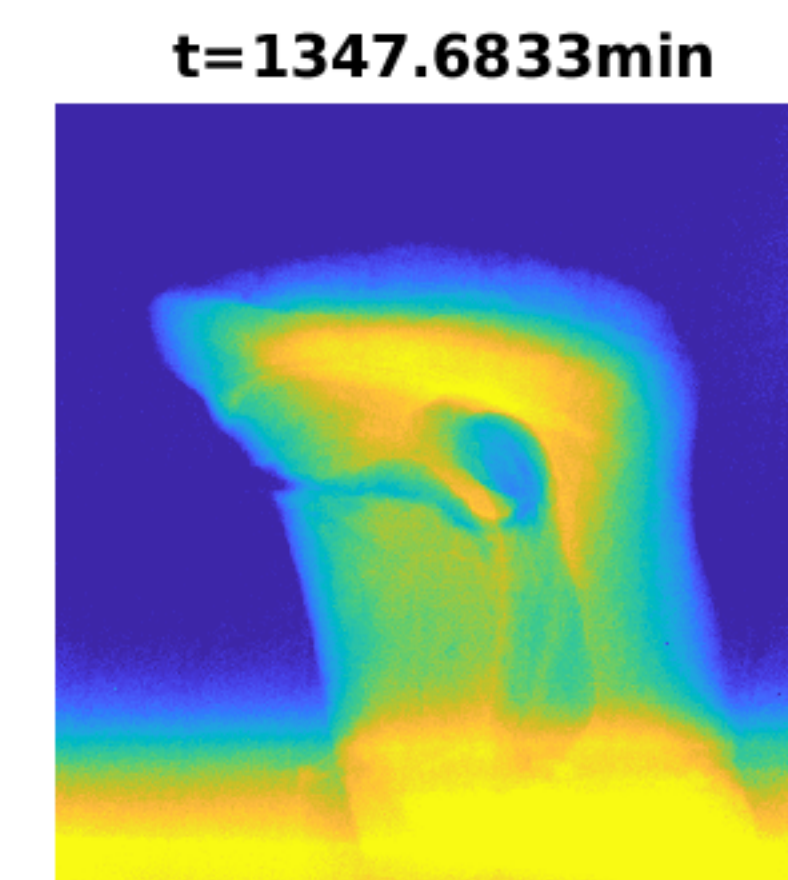

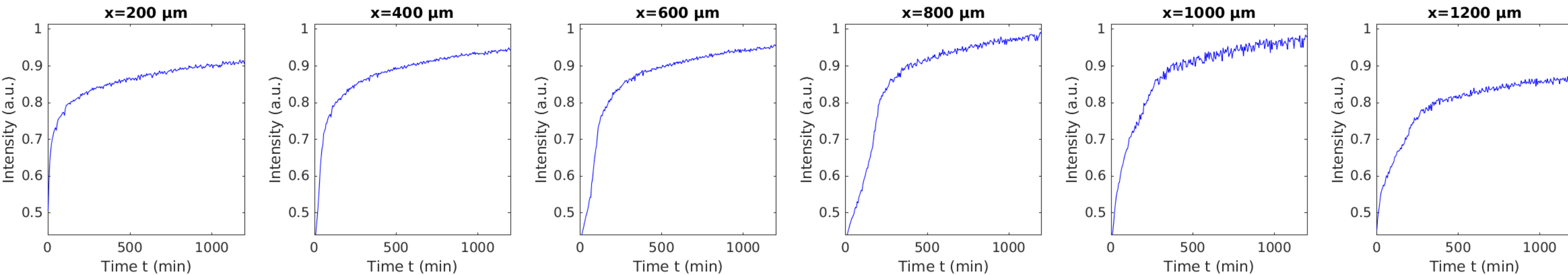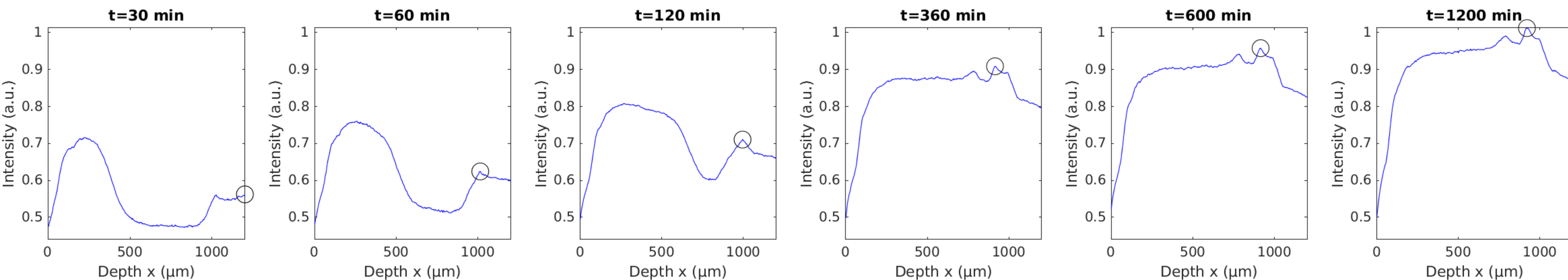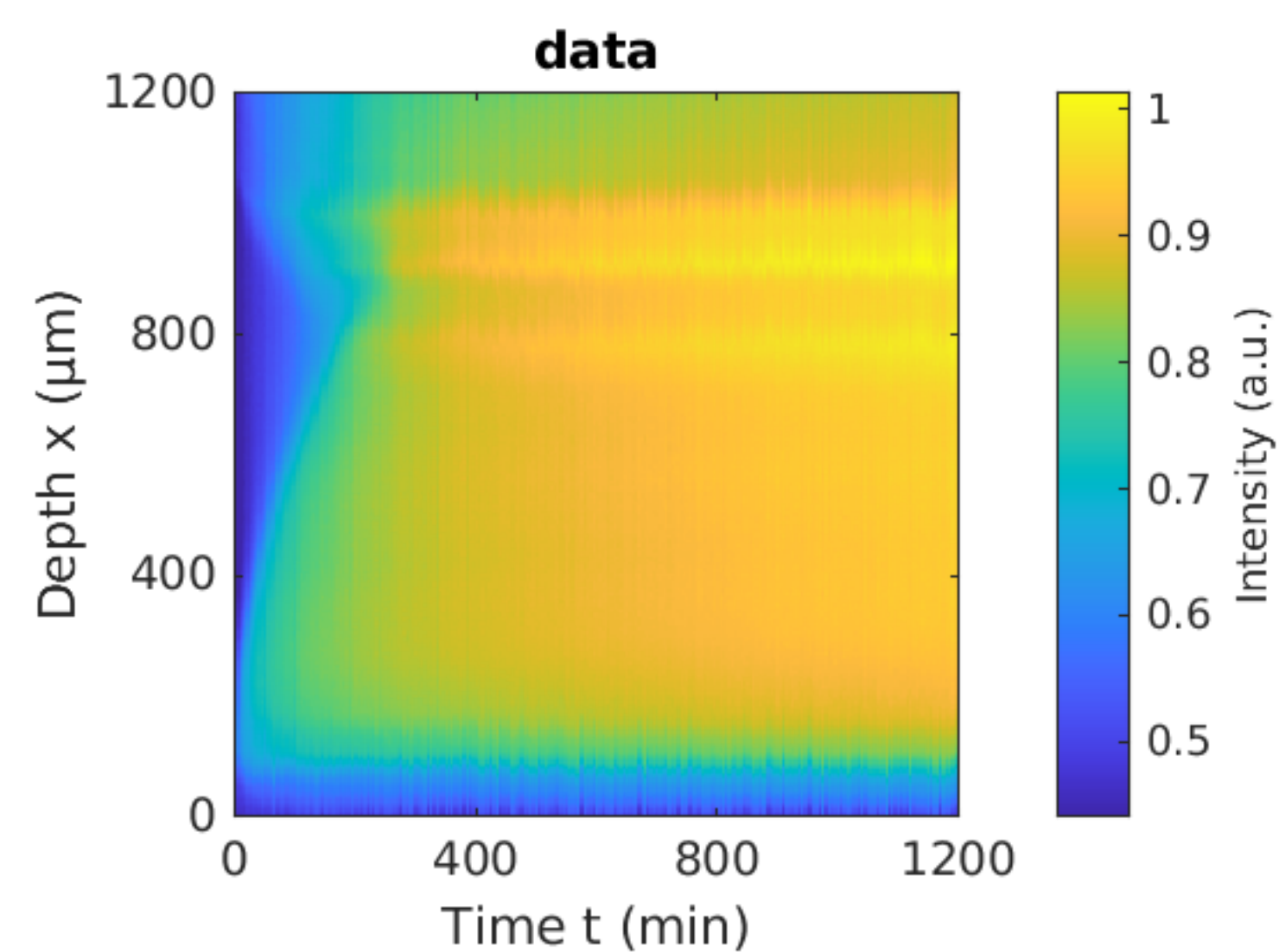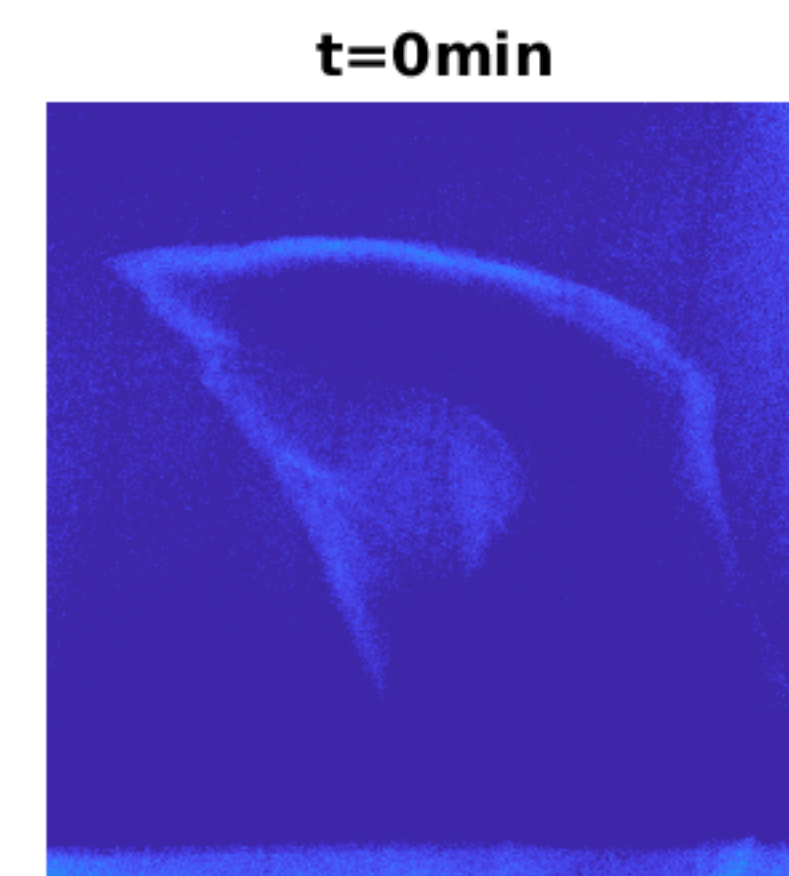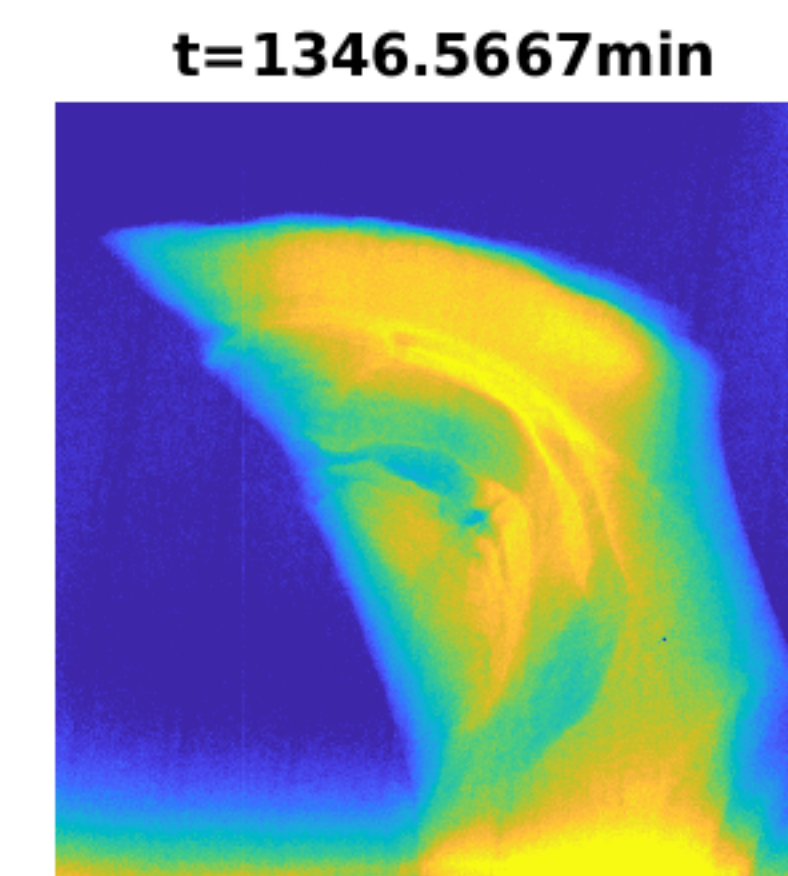

Supplement: Figure 1—source data 5. — The first row shows the temporal profile of osmium accumulation at different cortical depths, while the second row shows the spatial profile of osmium accumulation at different times after staining onset. The black circles indicate the location of axon tracts that tended to accumulate osmium faster and stronger. The third row shows the measured spatial-temporal accumulation of osmium (left) and the corresponding distribution of osmium in projection images of the sample at the beginning of the experiment and after about 22 hoursr of incubation (right). [file elife-72147-fig1-data5.pdf]

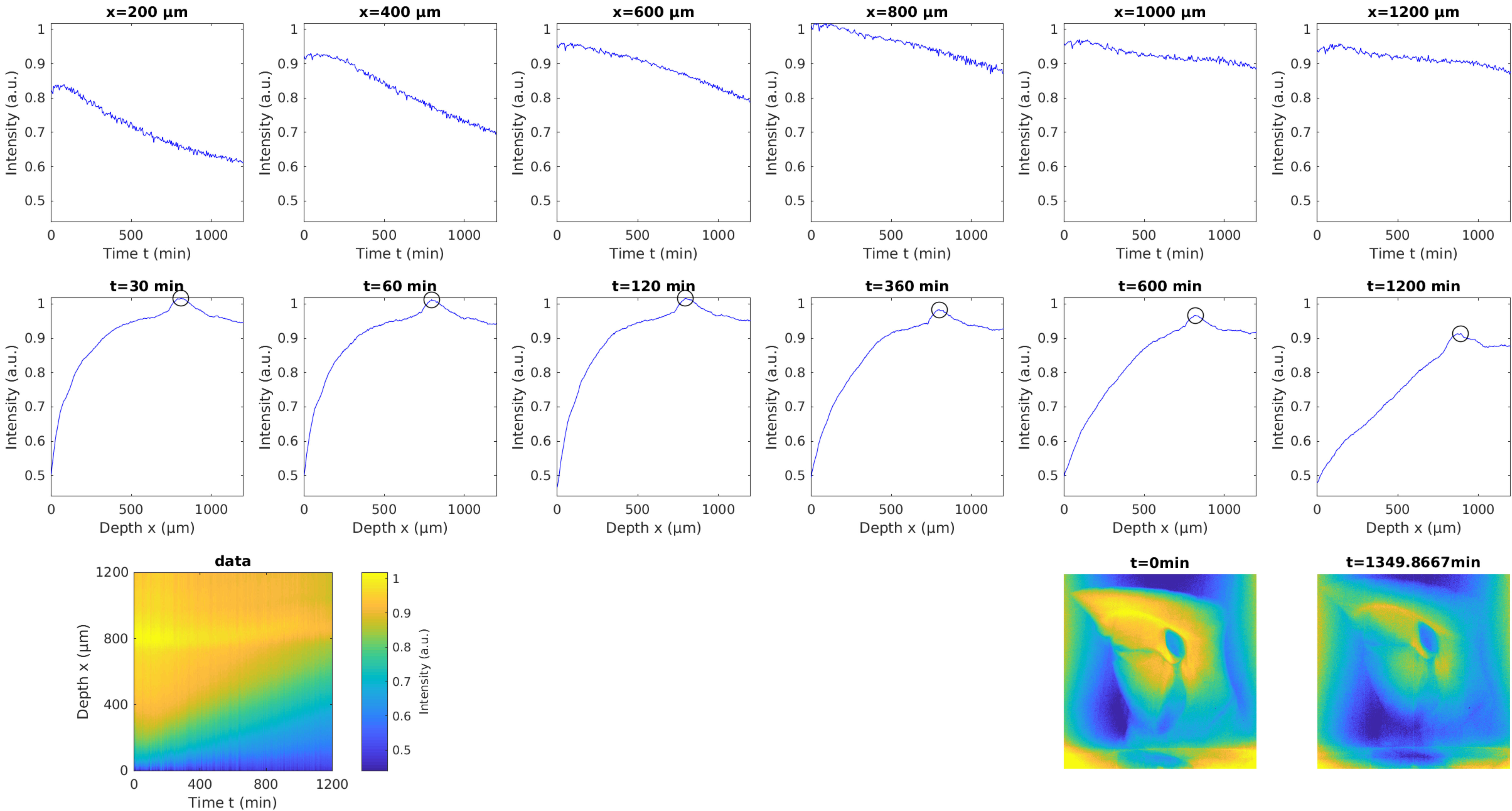

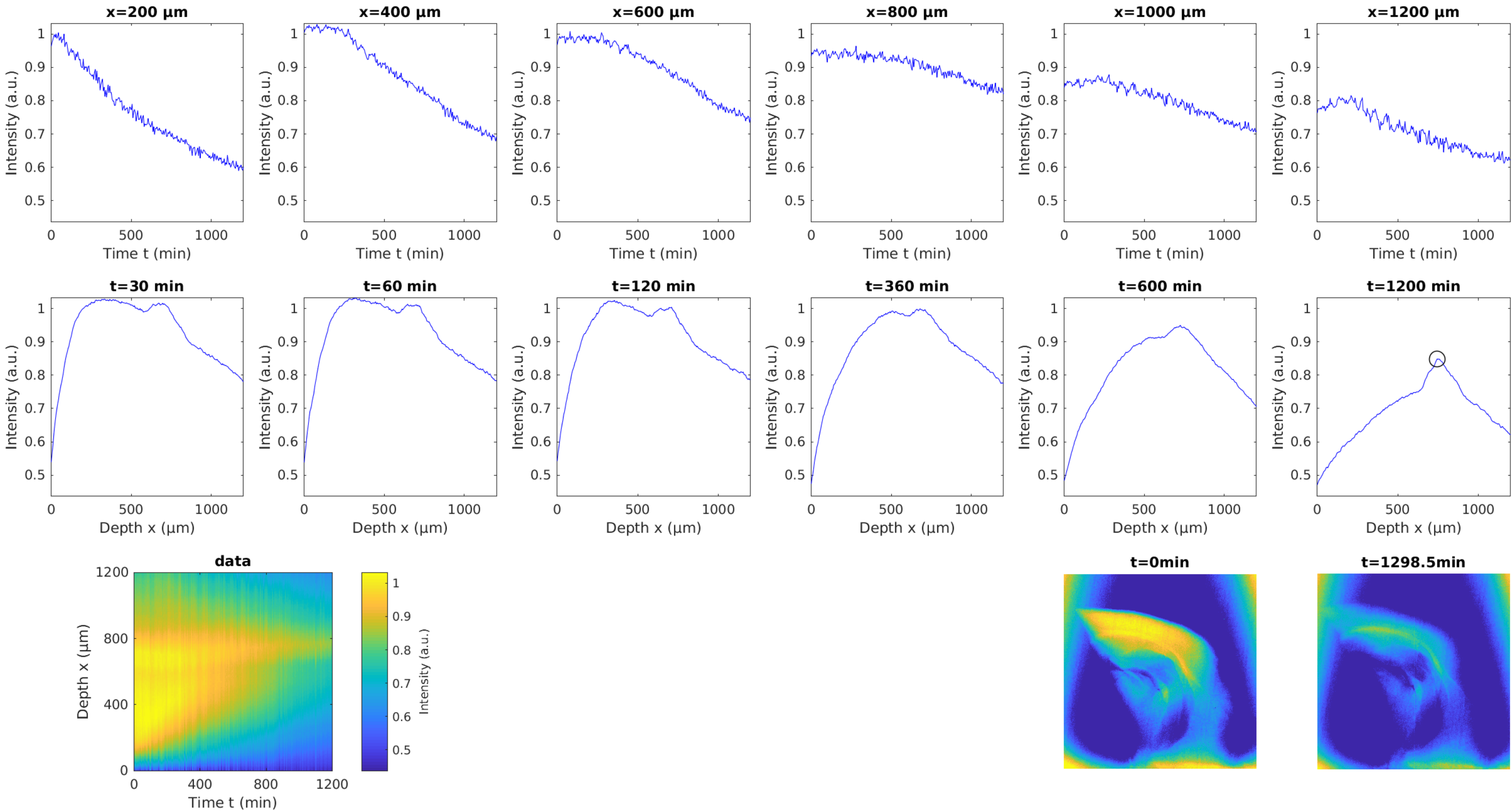

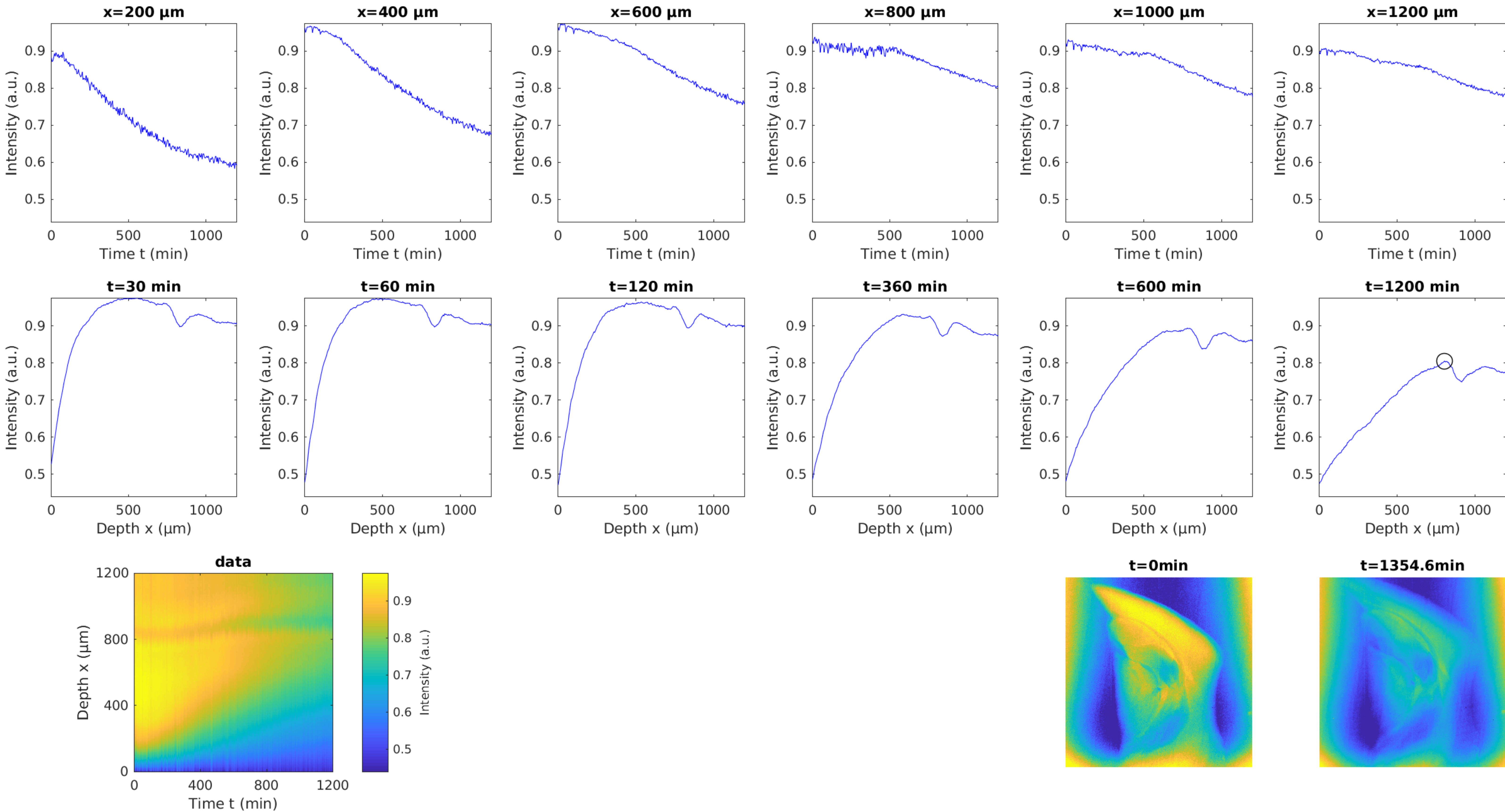

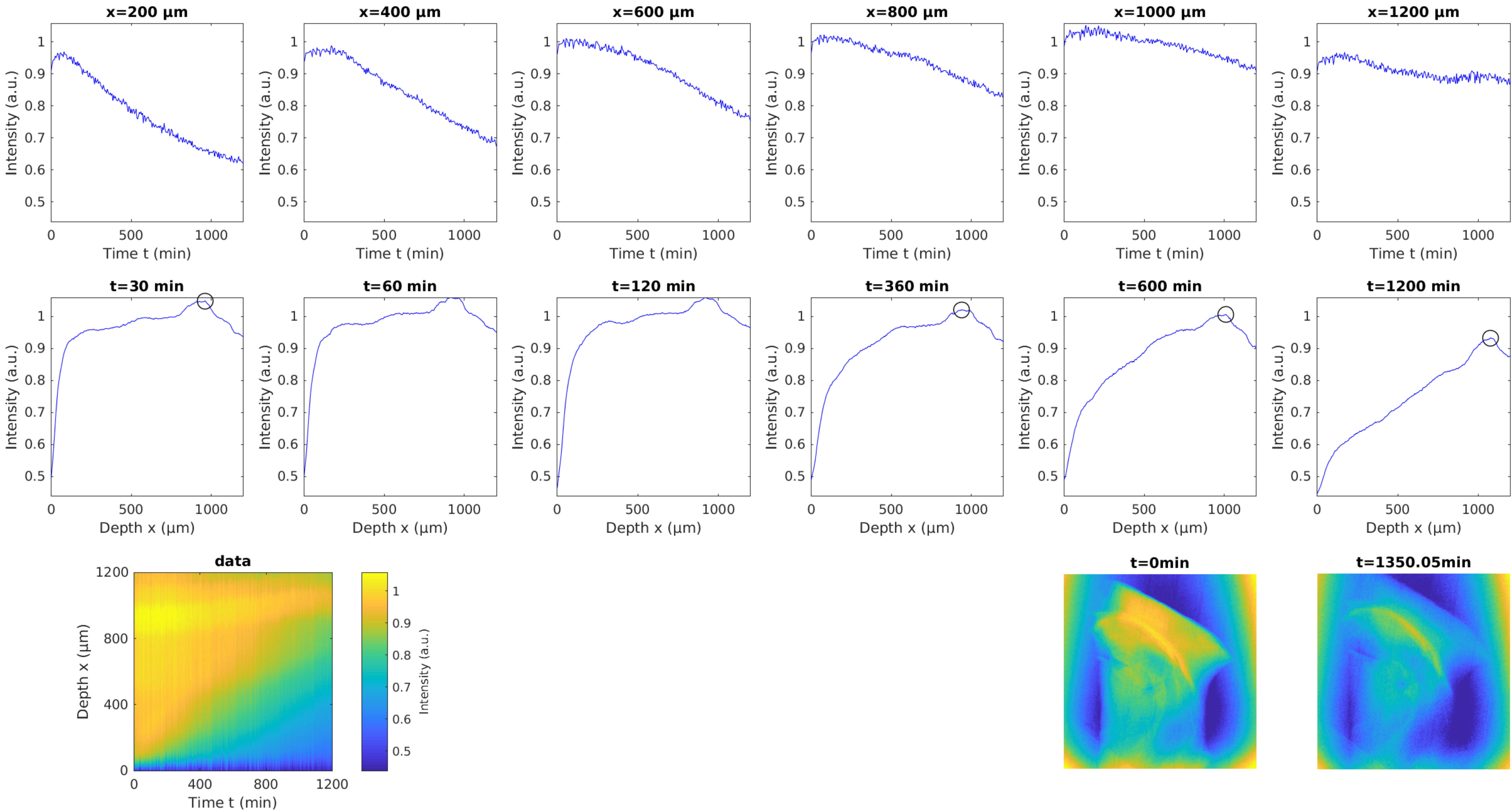

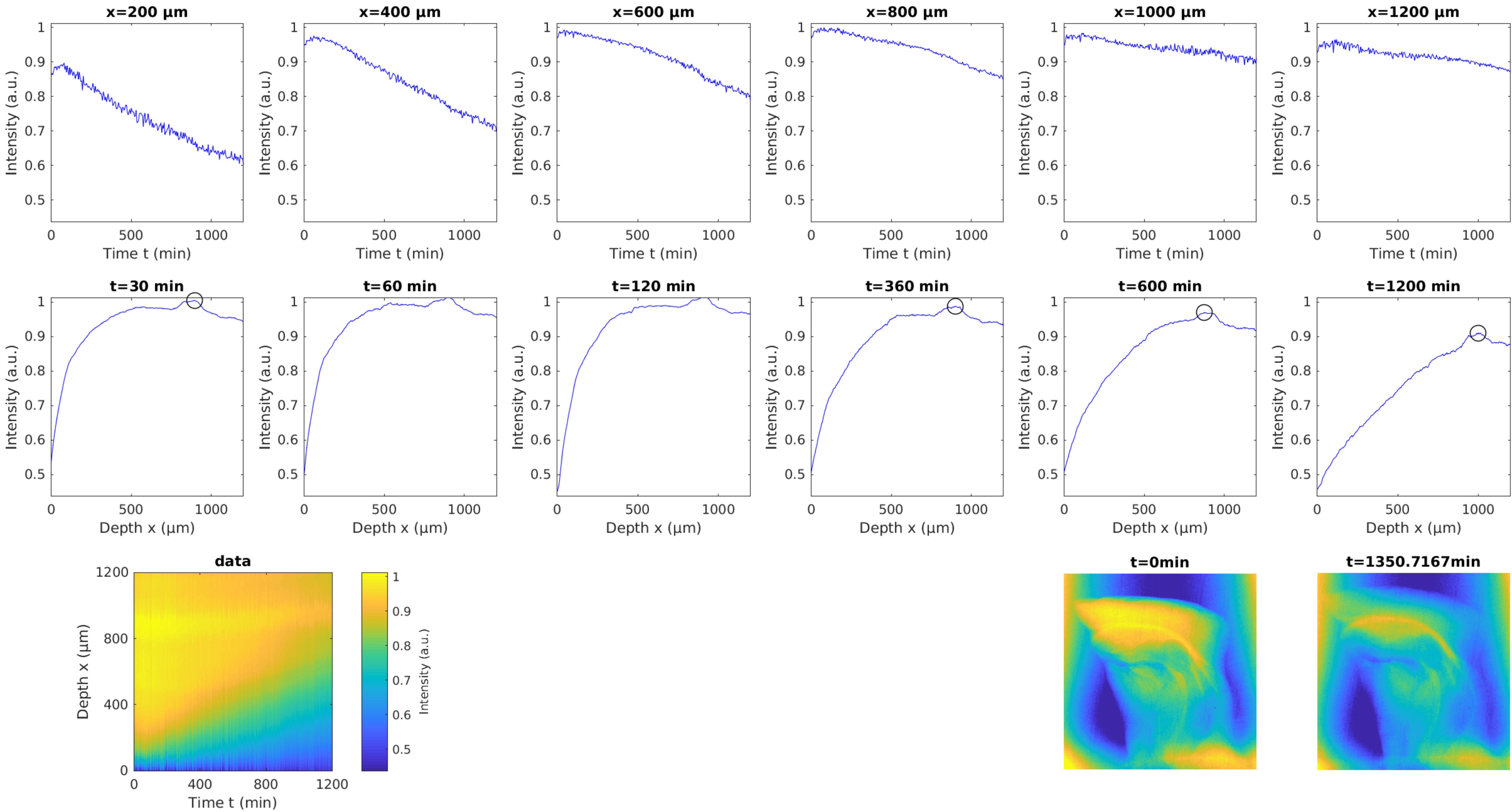

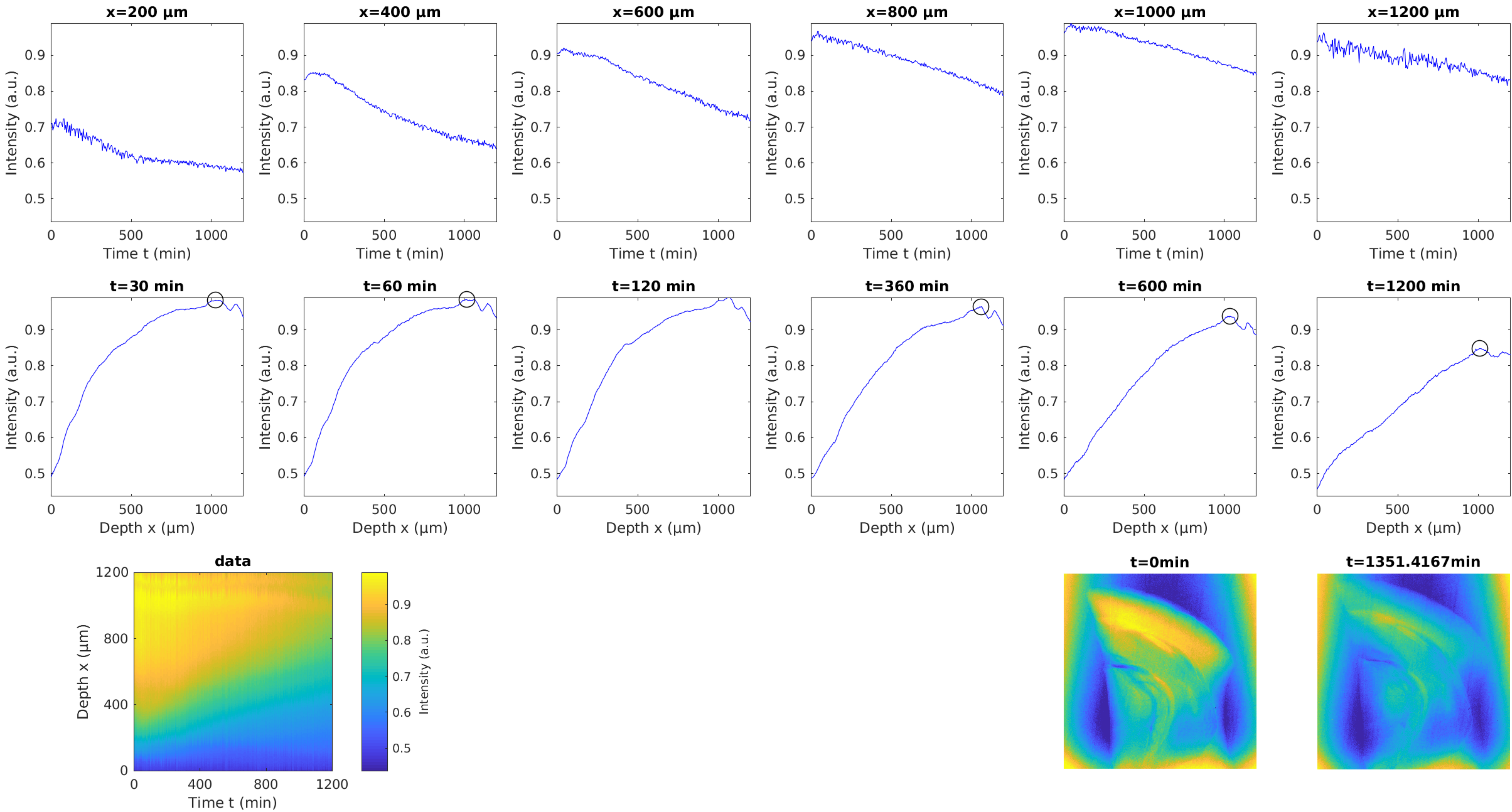

Supplement: Figure 2—source data 1. — The first row of every sample shows the temporal profile of osmium washout at different cortical depths, while the second row shows the spatial profile of osmium washout at different times after staining onset. The black circles indicate the location of axon tracts. The third row shows the measured spatial-temporal washout of osmium (left) and the corresponding distribution of osmium in projection images of the sample at the beginning of the experiment and after about 22 hr of incubation (right). [file elife-72147-fig2-data1.pdf]

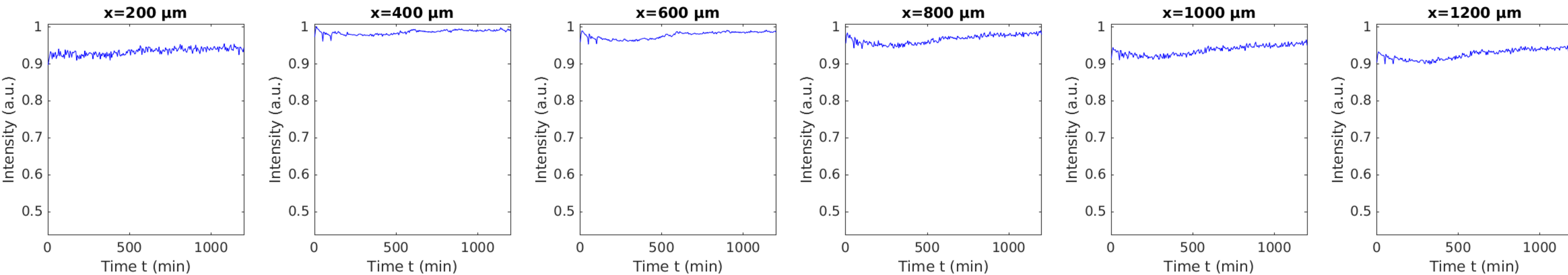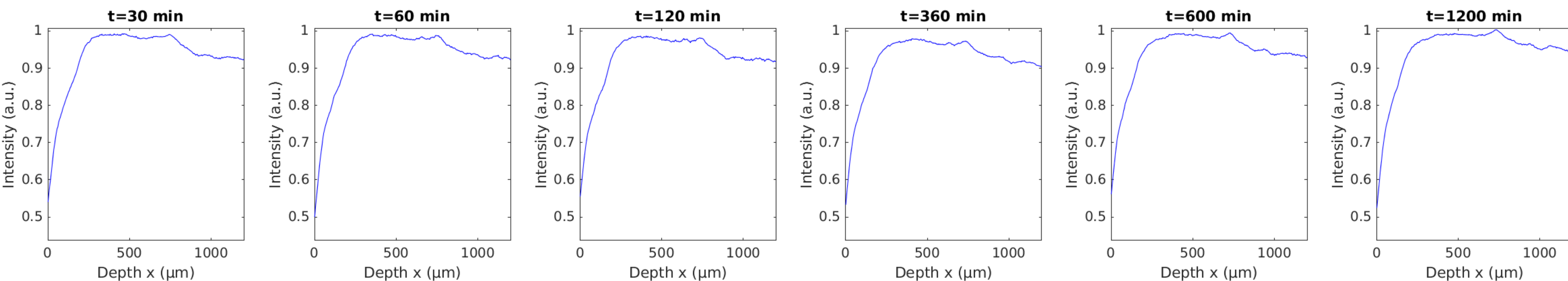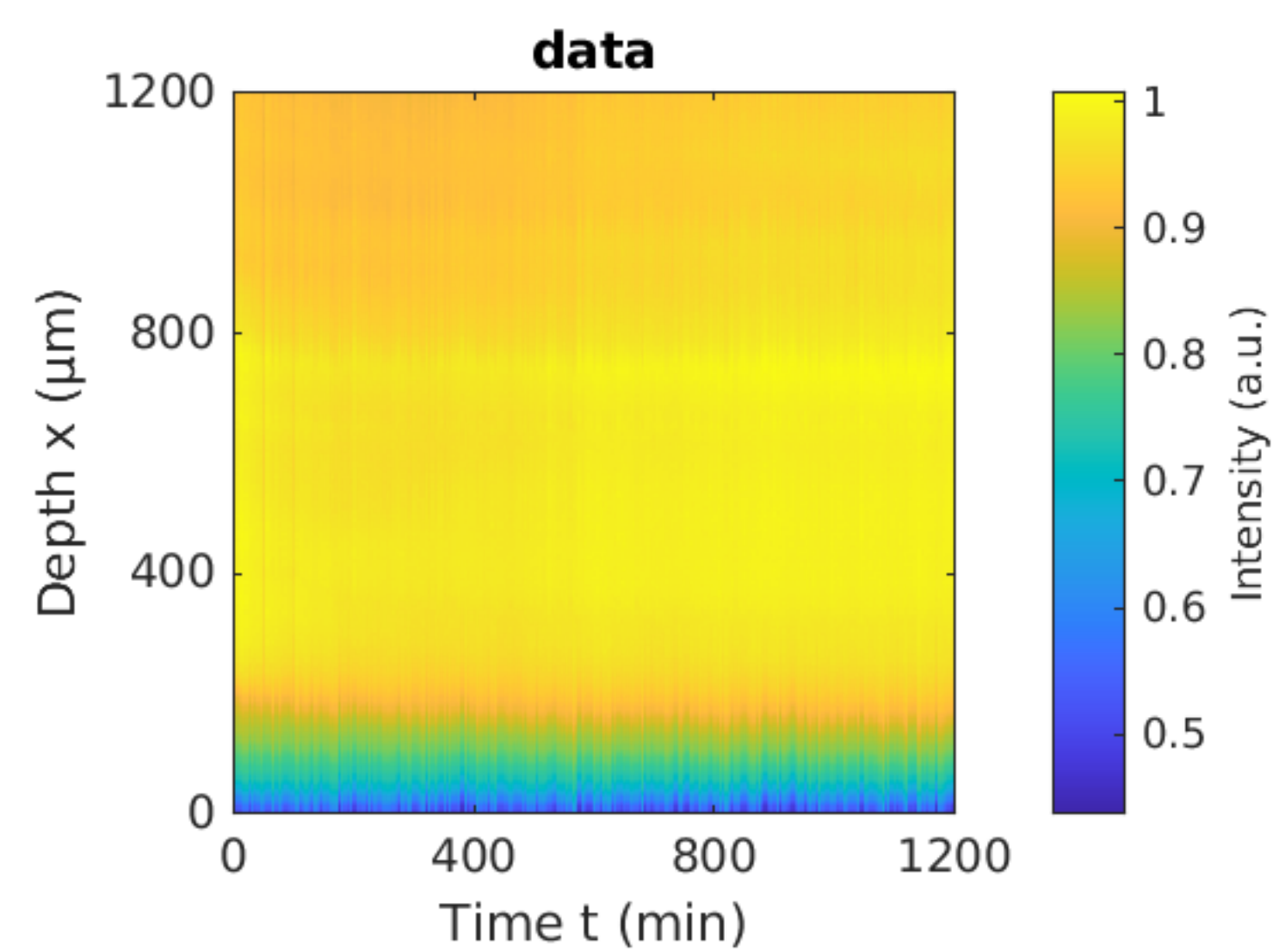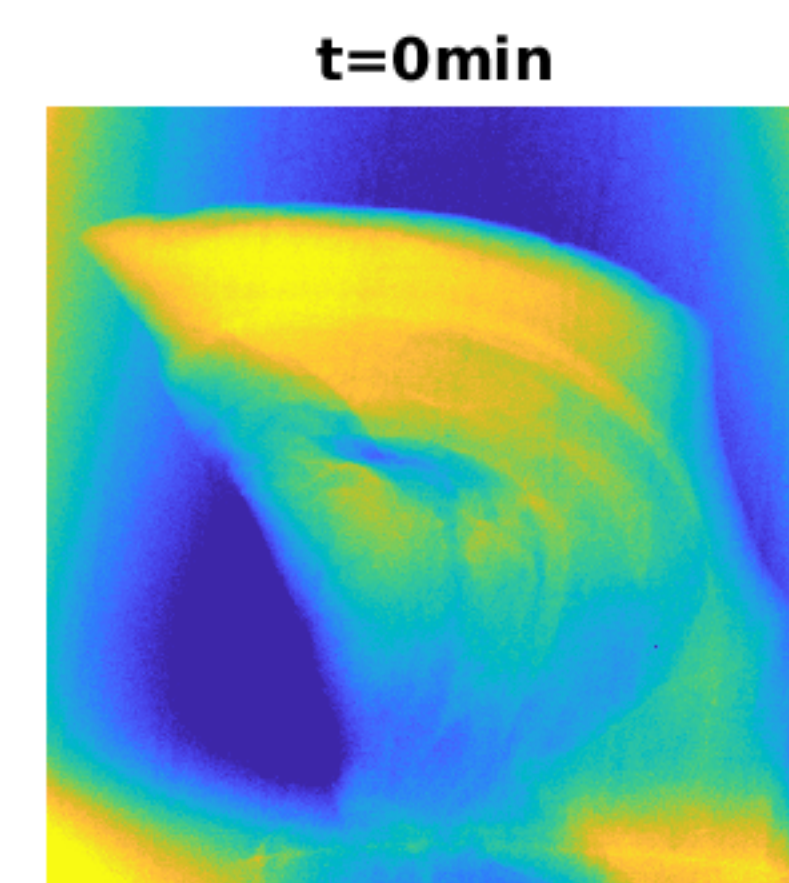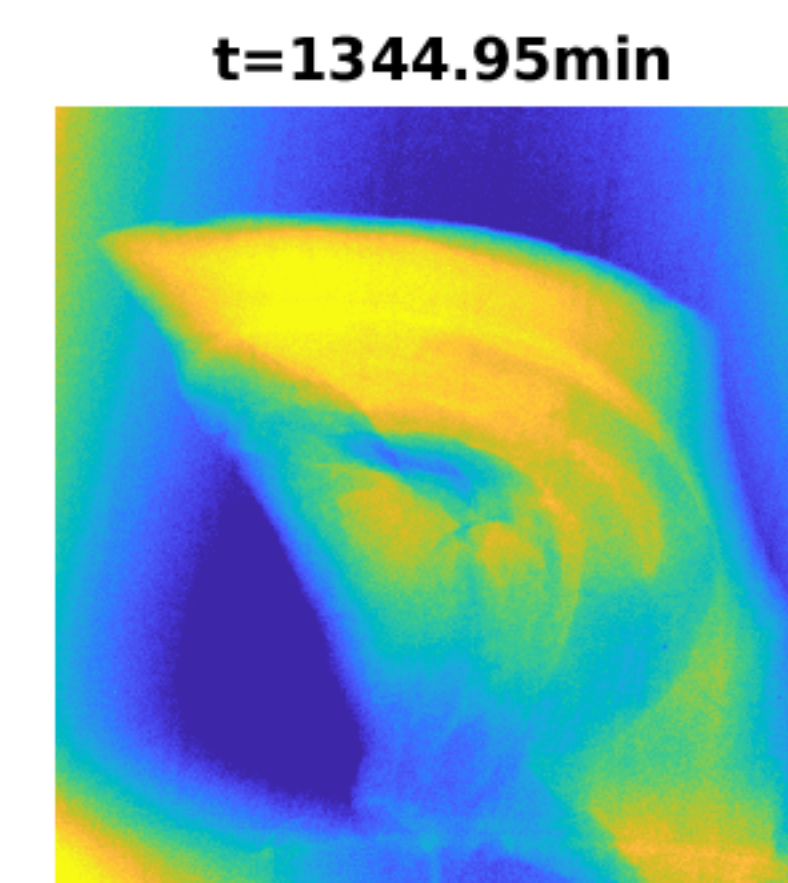

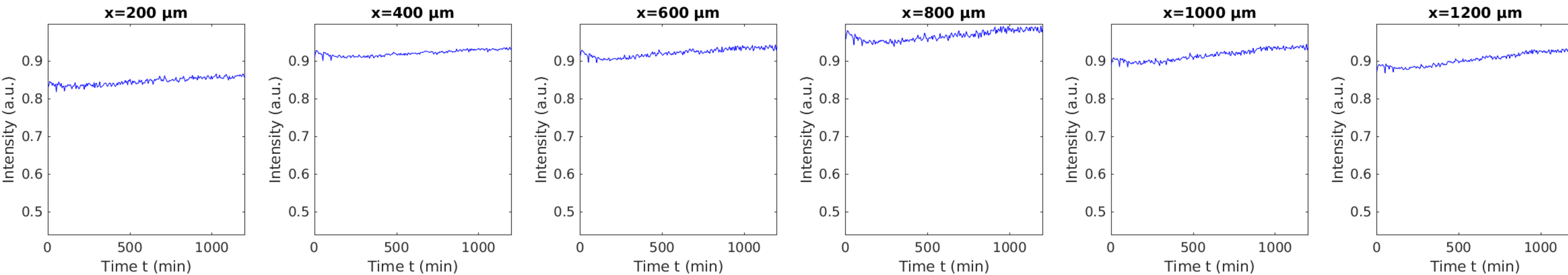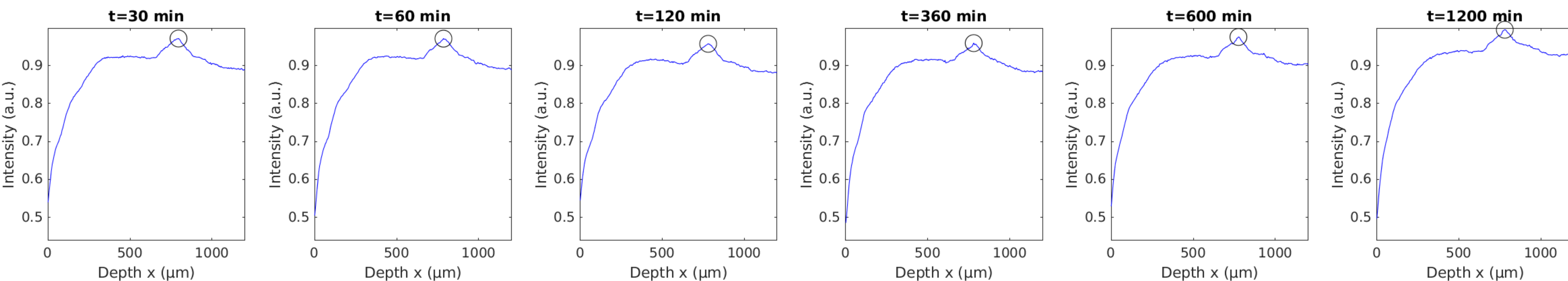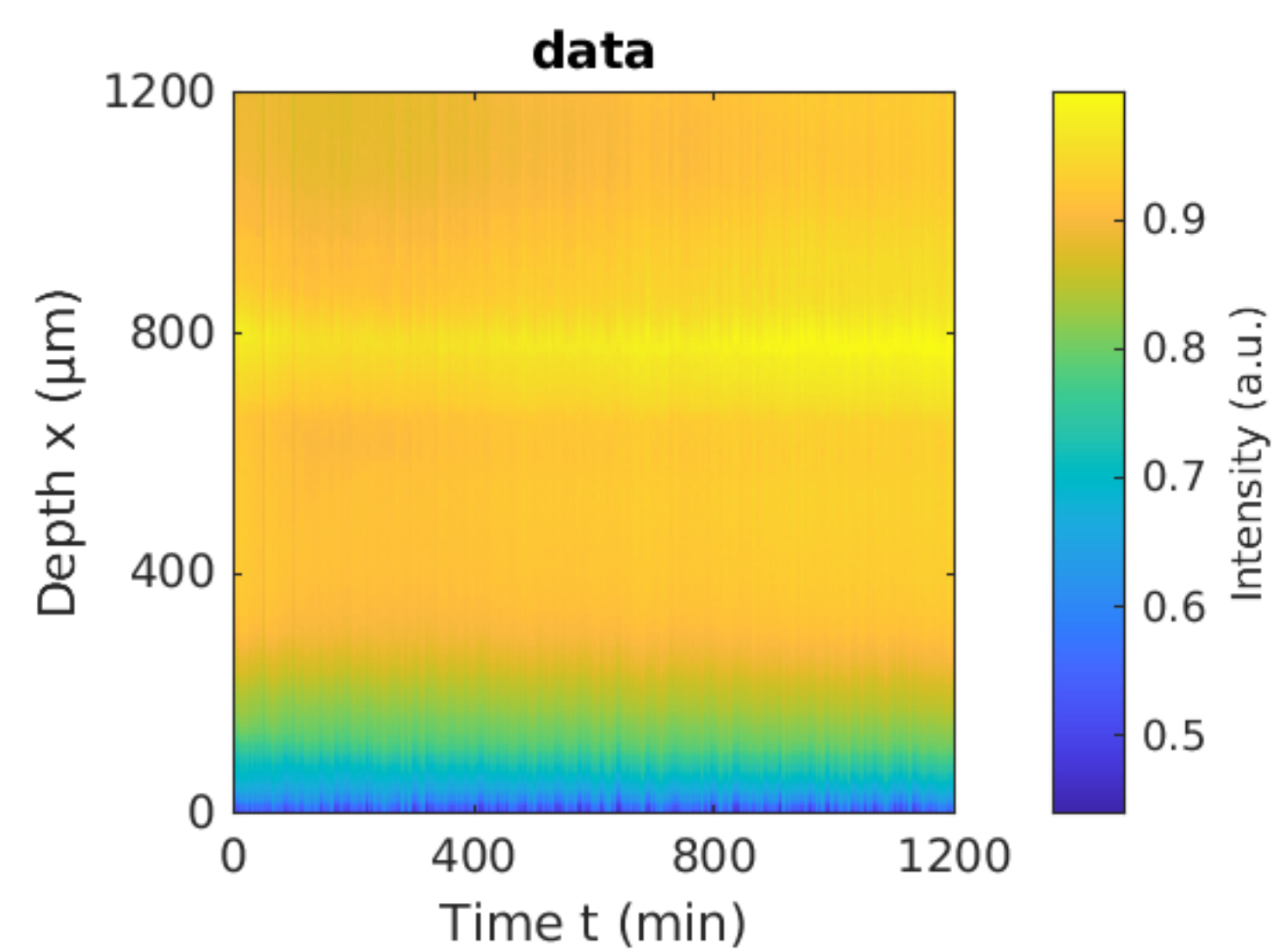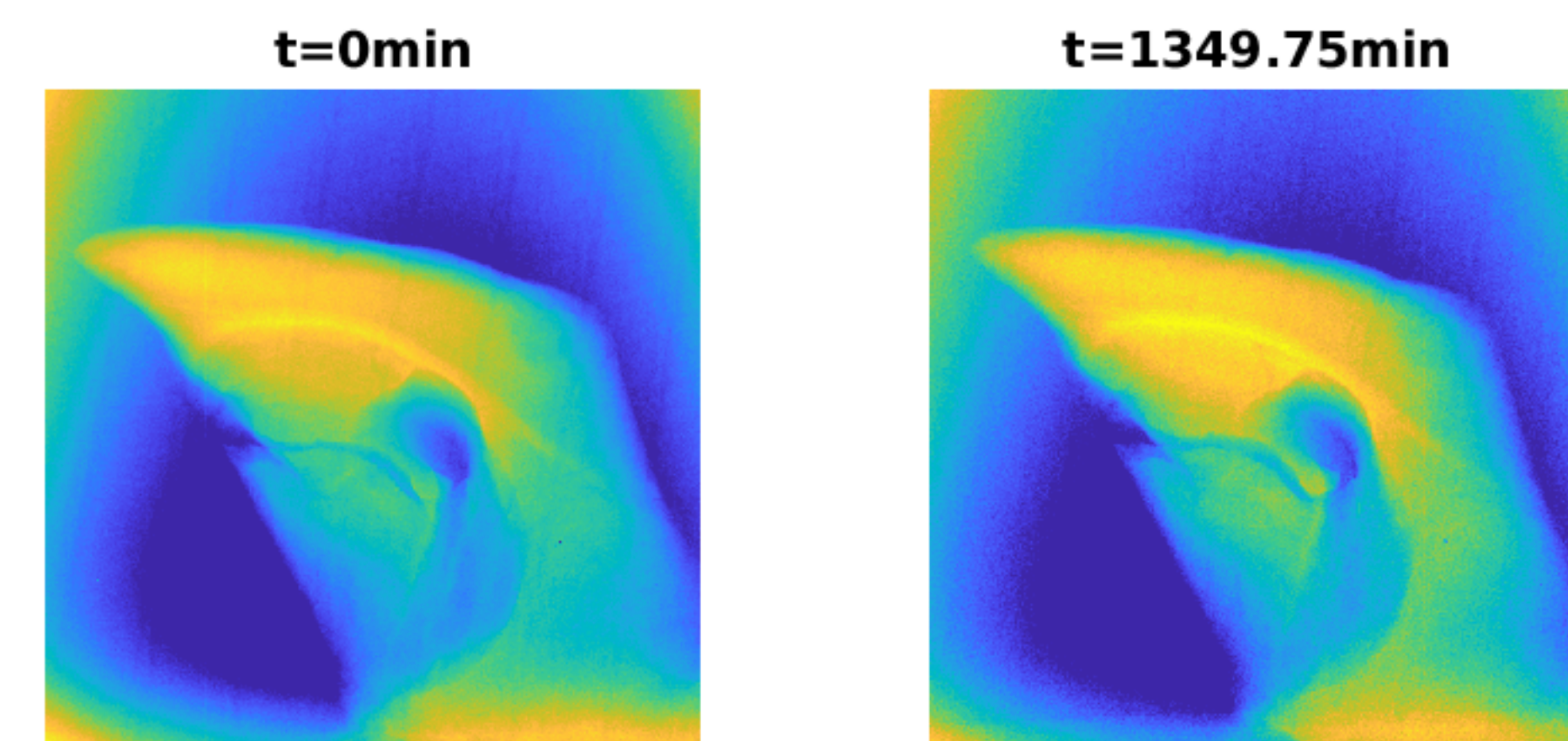

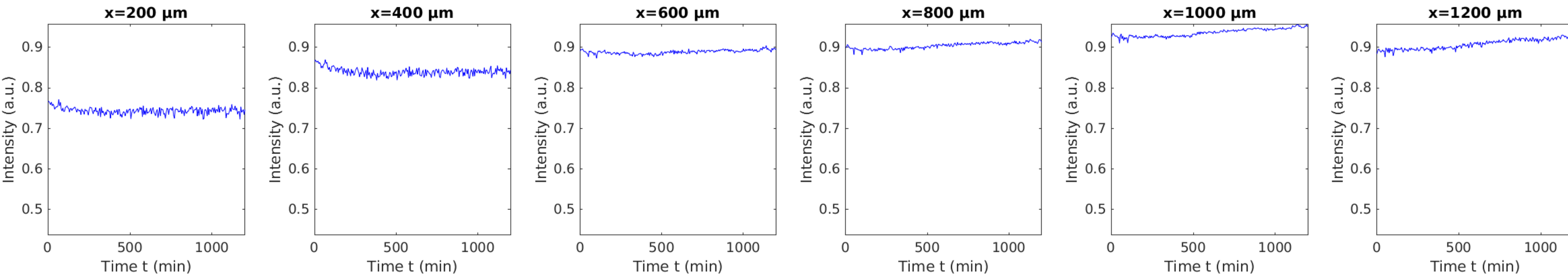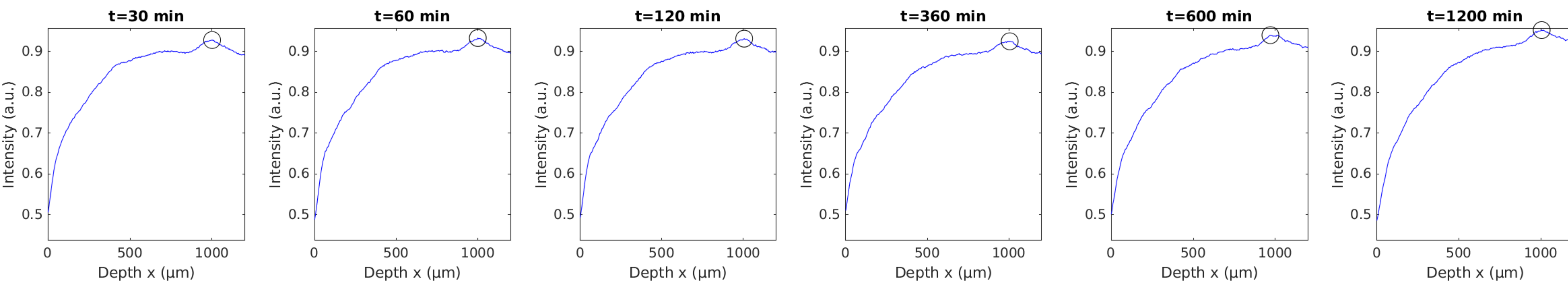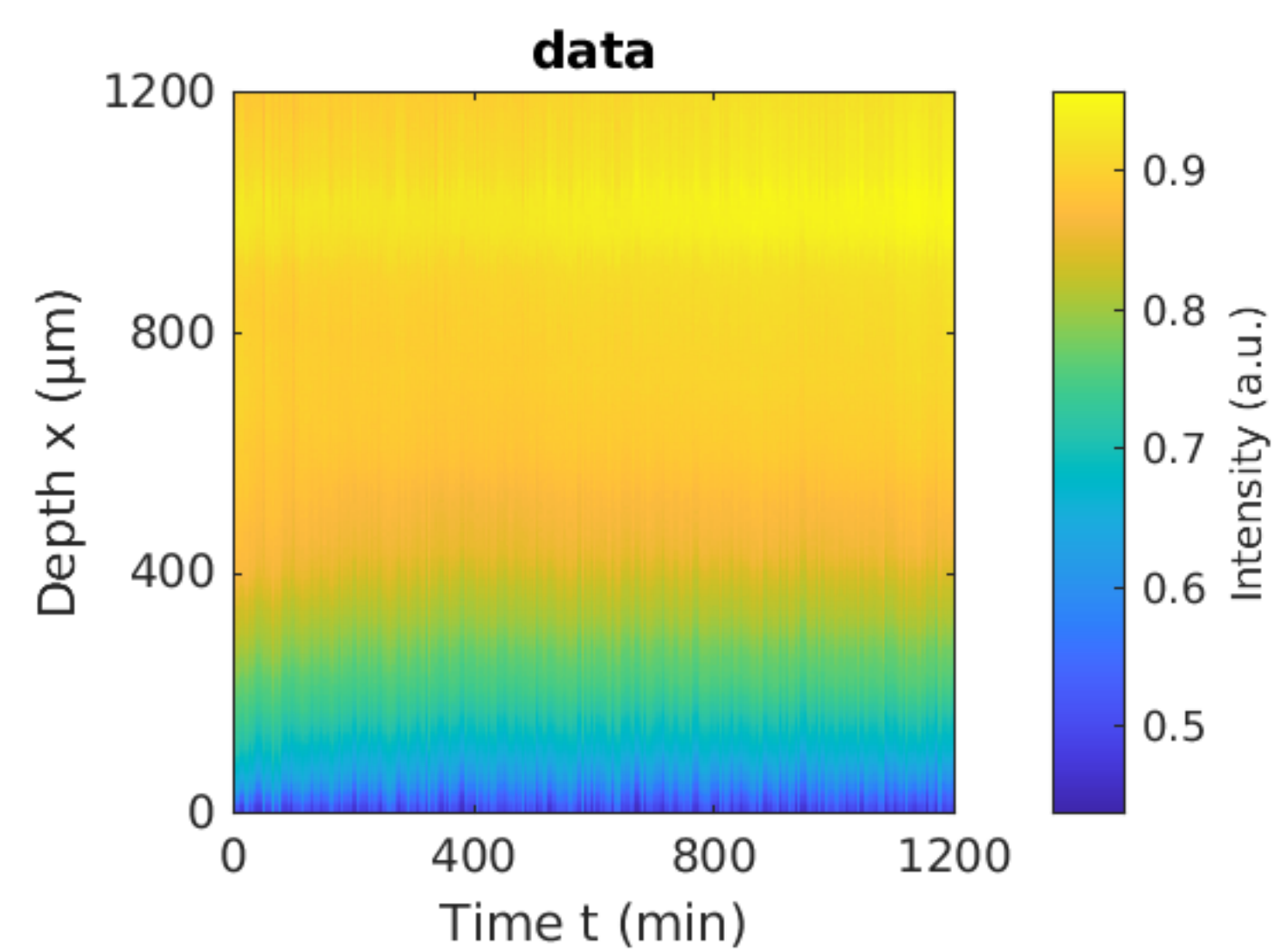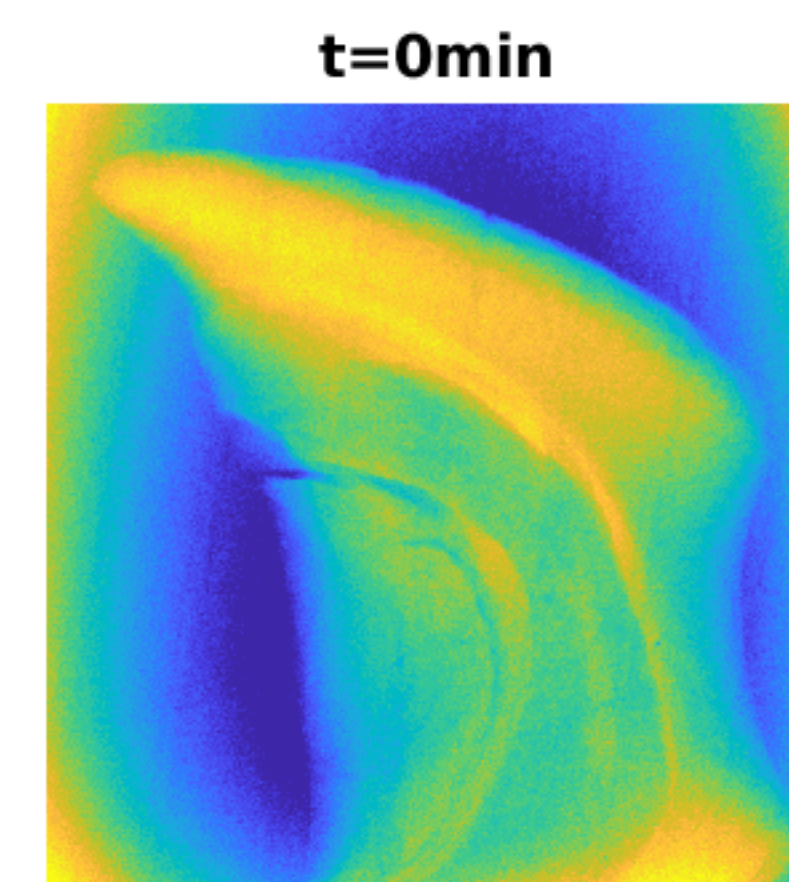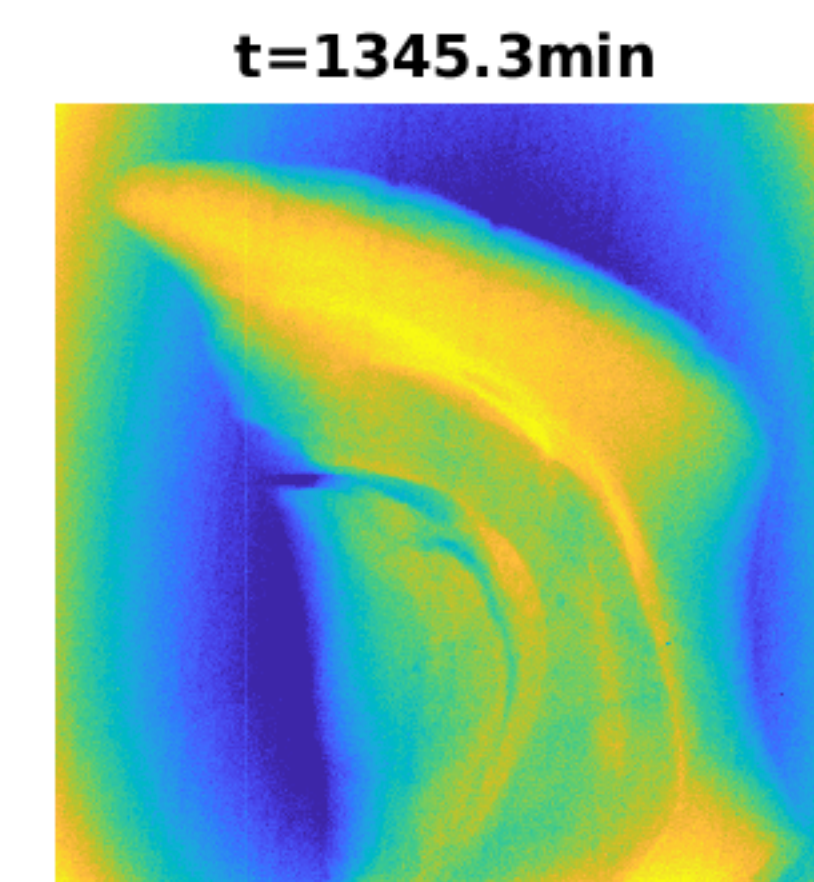

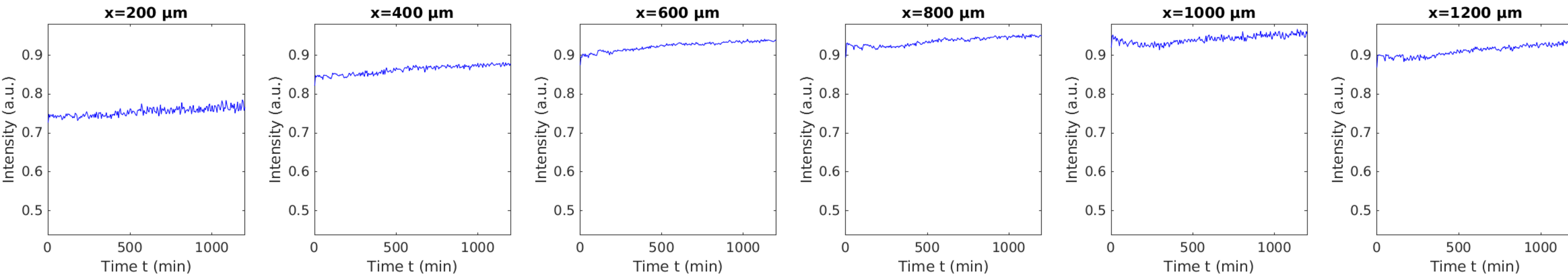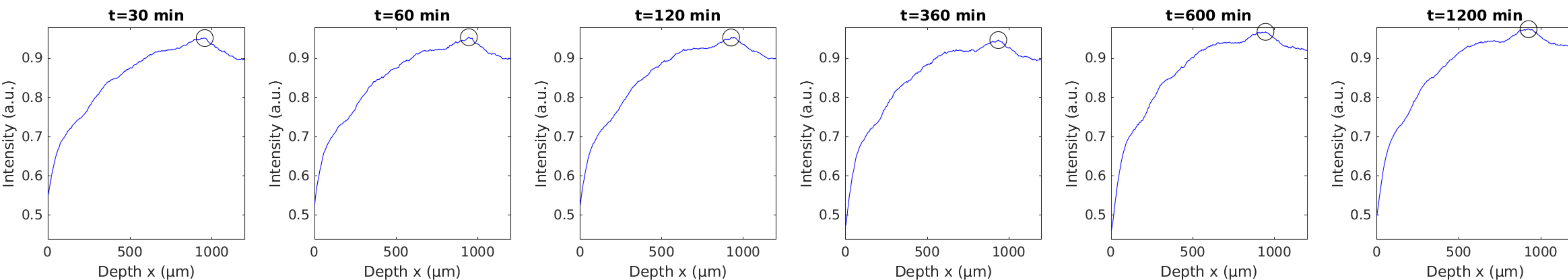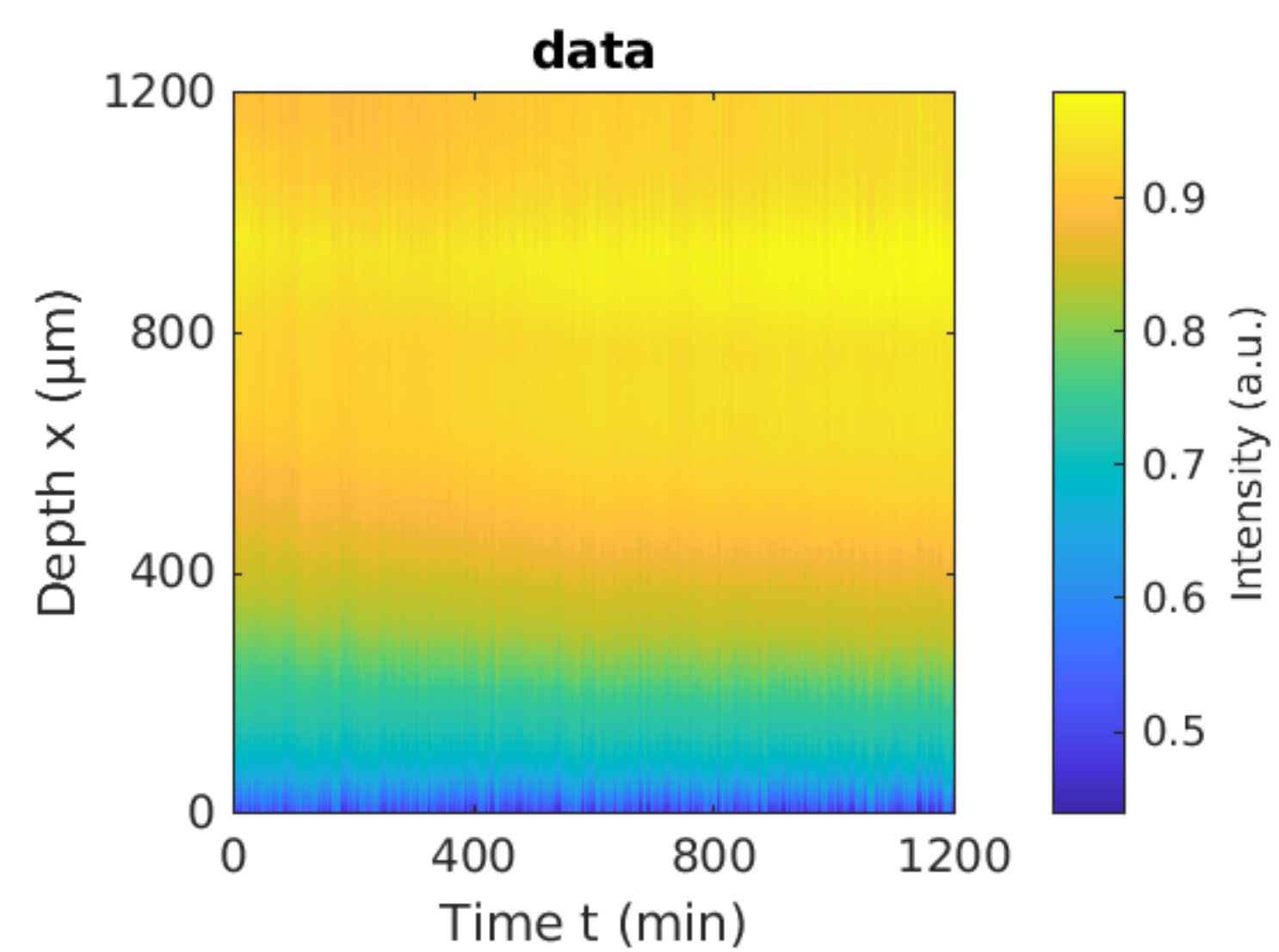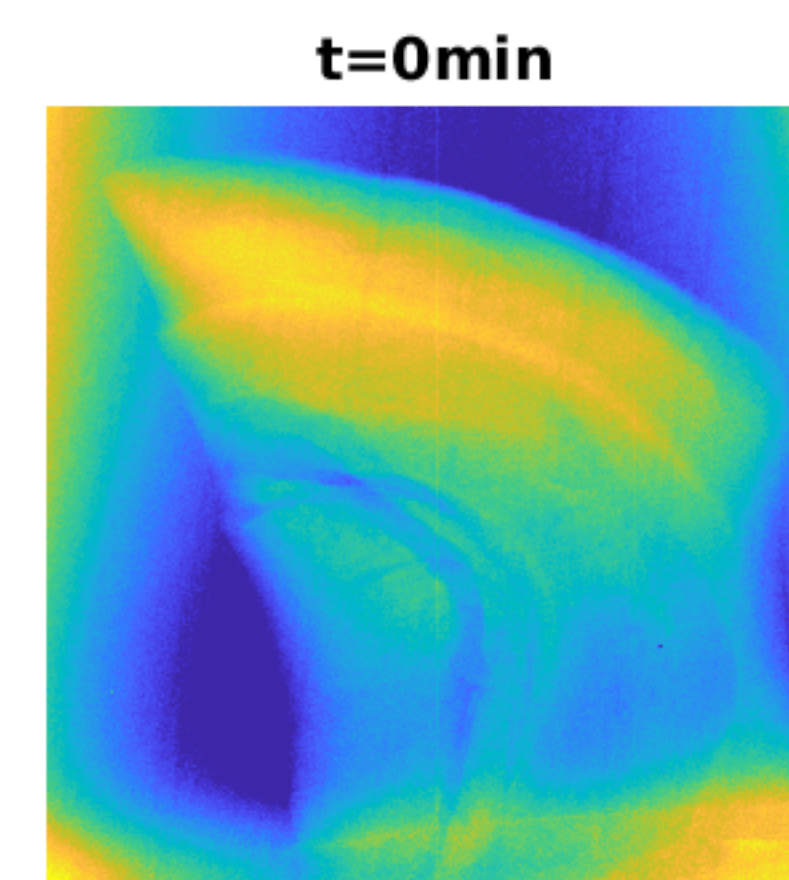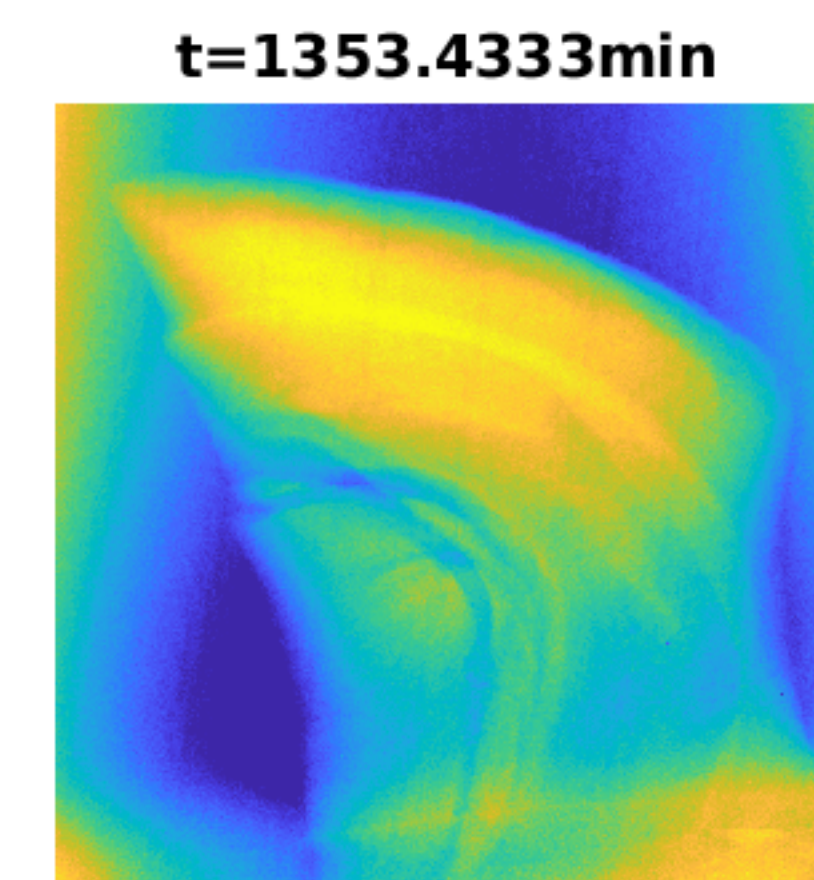

Supplement: Figure 2—source data 2. — The first row of every sample shows the temporal profile of osmium washout at different cortical depths, while the second row shows the spatial profile of osmium washout at different times after staining onset. The black circles indicate the location of axon tracts. The third row shows the measured spatial-temporal washout of osmium (left) and the corresponding distribution of osmium in projection images of the sample at the beginning of the experiment and after about 22 hoursr of incubation (right). [file elife-72147-fig2-data2.pdf]
